# The Impact of Essential Oils From Aromatic Plants on Microbial Dynamics and Nutrition in Lacto‐Fermented Systems

**DOI:** 10.1002/fsn3.70948

**Published:** 2025-12-18

**Authors:** Ibrahim Canbey, Tulay Ozcan, Ozan Gurbuz

**Affiliations:** ^1^ Department of Food Engineering Bursa Uludag University Bursa Turkiye; ^2^ Graduate School of Natural and Applied Sciences Bursa Uludag University Bursa Turkiye

**Keywords:** essential oils, lactic culture, probiotics, secondary metabolites

## Abstract

Lacto‐fermented food products, including fermented dairy products, cereal products, processed fruits and vegetables, and meat products, may seem like the right choices for healthy food categories. Probiotic products in this group are generally produced by adding probiotic bacteria (*Lactobacillus* and *Bifidobacterium*, etc.) to food matrices. But in this production method, the short shelf lives of microorganisms require the addition of some prebiotics or natural substances like essential oils (EOs) of some plants to formulations of synbiotic products. The EOs are the most commonly known secondary metabolites that are responsible for characteristic scents and also display important bioactive properties, like antibacterial, antifungal, anti‐inflammatory, and antioxidant effects in medicinal and aromatic plants. EOs extracted from various spices and plants exhibit potent inhibitory effects against a broad range of microorganisms. The combination of secondary metabolites with lactic acid bacteria in food products can enhance probiotic viability while inhibiting pathogens through antimicrobial activity. However, EOs may also adversely affect lactic culture development. Further research is needed to identify suitable plant species and determine safe application methods for EOs in probiotic and lacto‐fermented products.

## Graphical abstract

Bioactive compounds found in essential oils (EOs), which are obtained by distilling certain plants, have been shown to inhibit pathogenic microorganisms and also support the growth of probiotic bacteria. In this context, EO nutraceuticals have properties that can improve the shelf life and sensory properties of products.

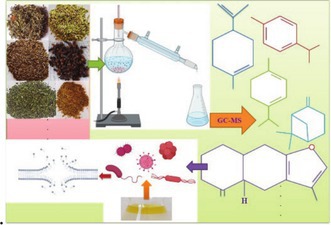

## 1 Introduction

In recent years, consumers who prefer a healthy lifestyle have turned to fermented functional foods containing natural ingredients (Shahbazi and Shavisi 2019). These healthy foods have great effects on the food industry, particularly fermented products like kefir, yogurt, cheese, pickled vegetables, dough breads, and sausages with nutritional components, including minerals, proteins, and vitamins (Massoud and Sharifan 2020; Vanegas‐Azuero and Gutiérrez 2018; Shiroodi et al. 2012).

Fermented foods also contain beneficial lactic acid bacteria (LAB) and probiotics. Probiotics, living microorganisms, are beneficial to the health of consumers (Massoud and Sharifan 2020). Besides, these microorganisms alleviate the responses of the immune system and increase macrobiotic activity in the gut and gastrointestinal functionality by regulating lactose intolerance (Mohajeri et al. 2021; Perina et al. 2015; Moritz et al. 2012; Ranadheera et al. 2010). In this regard, research on probiotics for the advantages of humans has concentrated on the new specific formulation in commercial and industrial areas, and some of them have even provided significant data on probiotics related to health and well‐being (Roobab et al. 2020; Mehdizadeh et al. 2019; Ghorbanzade et al. 2017).

Probiotics have beneficial effects on the health of consumers when used in adequate doses. The most commonly known bacteria considered for use in probiotic products are *Lactobacillus* and *Bifidobacterium*. These genera, such as *Lactobacillus casei* , *L. paracasei* , *L. acidophilus* (LA5), *L. fermentum* , *L. rhamnosus* , *L. plantarum* , *L. reuteri* , *Bifidobacterium bifidum* , *B. breve*, and *B. animalis* subsp. *Lactis* BB‐12 (*Bifidobacterium* BB‐12) can be added to products alone or in combination (Keshavarzi et al. 2021; Massoud and Sharifan 2020; Ahari et al. 2020; AL‐Saadi 2016; Azizkhani and Tooryan 2016). In probiotic products, maintaining viable cell counts at effective levels throughout storage until consumption is crucial (Eroglu and Ozcan 2024; Moritz et al. 2012). For beneficial and therapeutic effects, final products should contain at least 10^6^ CFU g^−1^, and the microorganisms must survive passage through the gastrointestinal tract (Mohajeri et al. 2021; Marinaki et al. 2016). According to many studies, the minimum probiotic concentration should be found in the final products at least 10^6^–10^7^ CFU/g or mL level to benefit from these health benefits (Mohajeri et al. 2021; Mehdizadeh et al. 2019; Azizkhani and Parsaeimehr 2018). Nevertheless, the survivability of the probiotics tends to decline depending on the previously mentioned factors, such as hydrogen peroxide, lower pH level, higher oxygen concentration, etc., and this situation is the major problem in maintaining the probiotics at the desired level, especially during storage (Narli and Ozcan 2022; Keshavarzi et al. 2021; Kim et al. 2019; Sarvari et al. 2014). Besides storage conditions, the other substantial factors in the reduction of probiotic viability in the host are a lower pH level in the stomach and also bile salts in the intestines (Eroglu and Ozcan 2024). In connection with this information, much research has been carried out on probiotic survivability, especially under bile salts produced by the small intestine (Sahadeva et al. 2011), higher acidic levels of the stomach, and additionally in foods stored in cold conditions (Azizkhani and Parsaeimehr 2018; Fazilah et al. 2018). However, unfortunately, in the products produced with probiotics like *Lactobacilli* and also *Bifidobacterium*, the survivability of these bacteria can be reduced, especially during storage (Massoud et al. 2015; Mehdizadeh et al. 2019; Ahari et al. 2020; Massoud and Sharifan 2020). Especially in dairy products, the decrease in pH (~4.2) due to the activation of the beta‐galactosidase enzyme during storage at 0°C–5°C negatively affects the number of live probiotic bacteria due to the increased hydrogen ions compared to lactate ions (Kailasapathy 2006; Ozdemir and Ozcan 2020). The undesired lower pH values (less than 4) cause the activation of lactic cultures, especially *Lactobacillus delbrueckii* subsp. *bulgaricus*, excessively, resulting in more proteolytic activity and a large amount of acetaldehyde, lactic acid, and other organic acids formation (Ozcan et al. 2021; Mahmoudi et al. 2014). Besides, producers and researchers together try to find new advancements by combining various natural plant materials like essential oils (EOs) with probiotics (Mohajeri et al. 2021; Lucatto et al. 2020; Khaledabad et al. 2020). In this regard, to promote lactic and probiotic bacteria growth and kill pathogens (e.g., *Staphylococcus aureus* , *Escherichia coli* O157, etc.), new applications are being tried, like using natural plant metabolites, such as EOs. Volatile organic compounds extracted from botanical sources, commonly referred to as EOs, are intricate blends of hydrophobic secondary metabolites (Lappa et al. 2017; Oüzek et al. 2017). Extensive research has been conducted on the antimicrobial properties of EOs. For instance, oregano‐derived EO has demonstrated significant efficacy against a broad spectrum of microbial species, including fungal pathogens such as *Candida albicans* and both Gram (+) and Gram (−) bacteria (Leyva‐López et al. 2017; Adame‐Gallegos et al. 2016). The precise mechanism underlying the antimicrobial effects of EOs remains incompletely elucidated, largely due to the complexity of their chemical composition. Rather than acting through a single, well‐defined biochemical pathway, it is hypothesized that their antimicrobial potency results from interactions with multiple cellular structures and processes (Andrade‐Ochoa et al. 2021; D'Agostino et al. 2019). Predominantly, EOs are thought to disrupt microbial viability by altering membrane integrity, interfering with electron flow, disrupting ion gradients, impairing protein translocation, modifying phosphorylation pathways, and affecting enzymatic reactions dependent on these processes. Certain investigations suggest that monoterpenes can integrate into phospholipid bilayers, leading to structural perturbations in the membrane, whereby EO constituents function as disruptive agents within the organized lipid framework (Andrade‐Ochoa et al. 2021). In this study, the effect of EOs of aromatic plants on microbial dynamics of lacto‐fermented systems was investigated.

## 2 The Essential Oils

Many foods are inherently susceptible to spoilage during production, storage, marketing, and distribution. Therefore, the foods should be protected to procure the desired properties and shelf life. One of the major spoilage parameters in foods is undesired microorganisms, such as spoilage bacteria and fungi. These microorganisms contribute to the deterioration of sensorial and textural properties of foods, such as changes and losses in color, flavor, odor, etc. In addition to the spoilage problem, foodborne illness can emerge from eating food contaminated with pathogens like *Bacillus cereus* , *Clostridium perfringens* , *E. coli* O157, fecal and total coliforms, *Listeria monocytogenes* , *S. aureus* , *Salmonella* spp., *Vibrio parahaemolyticus* , molds, and yeasts. Food manufacturers and researchers are trying to reduce the increase in foodborne diseases caused by these microorganisms. These pathogens cause food poisoning infections in over 90% and lead to critical dangerous clinical problems (Andrade‐Ochoa et al. 2021; Mohamed et al. 2013). Considering these problems, several preservation methods, including acidification, drying, heat treatment, salting, etc., are applied to foods. Synthetic agents and chemicals can also be added to products to prohibit the growth of pathogenic and spoilage microorganisms. However, negative effects of these chemical additives on health lead researchers to find new natural materials. In recent years, the application of EOs has been of great interest in the food industry due to increasing consumer and legal authorities to kill pathogens, extend the shelf life of the products, and also procure desired organoleptic and functional properties to foods (Alayoubi et al. 2020; Mehdizadeh et al. 2019, 2018; Mohamed et al. 2013; Soković et al. 2010; Arques et al. 2008).

EOs are highly significant secondary metabolites found in numerous medicinal and aromatic plants, imparting distinctive fragrances to their botanical sources (Fikri et al. 2020). These secondary metabolites are complex blends of volatile, hydrophobic organic compounds, customarily extracted through steam distillation from various botanical sources, including fruits, blossoms, foliage, stems, seeds, fragrant woods, and roots. These substances generally embody the characteristic aroma of the plant from which they are derived (Matera et al. 2023). These substances typically exist as liquid extracts, possess a lower density than water, emit a strong and pervasive aroma reminiscent of their plant origin, and appear either colorless or slightly yellow with a translucent consistency. Within plants, EOs serve diverse biological roles: they facilitate pollination, function as storage compounds, contribute to allelopathic interactions by influencing the growth of neighboring plants, act as protective agents against specific insect species, and exhibit both wound‐healing and antimicrobial properties (Burt 2004). The classification of EOs is determined by multiple factors, including their physical state, botanical source, and the chemical composition of their predominant constituents (Andrade‐Ochoa et al. 2021; Fikri et al. 2020).

In addition, EOs are at times highly intricate blends of volatile, nonpolar or mildly polar compounds exhibiting diverse structural characteristics. Nevertheless, these constituents can be categorized into groups sharing intrinsic structural resemblance and a common biosynthetic pathway. A significant proportion of these molecules is hydrocarbons, composed solely of carbon and hydrogen, whereas others incorporate oxygen (commonly termed oxygenated compounds) and, in rarer cases, nitrogen or sulfur. However, classification based on elemental composition is less informative than grouping them according to their biosynthetic origin, which fundamentally determines their structural framework. In this regard, the majority of typical EO constituents fall into one of two principal categories: terpenoids and phenylpropanoids (Matera et al. 2023). Terpenes, along with their oxygen‐containing derivatives known as terpenoids, represent the predominant constituents of EOs (Rehman et al. 2016), whereas phenylpropanoids and benzenoid compounds are present in comparatively lower concentrations (Fikri et al. 2020).

**FIGURE 1:**
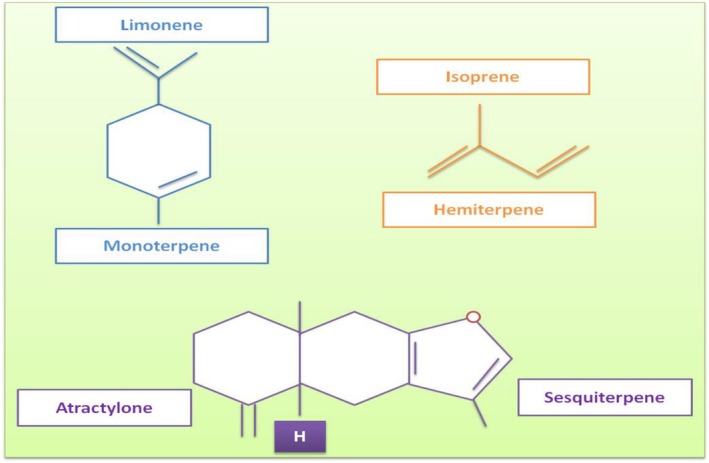
The chemical structures of monoterpene limonene, hemiterpene isoprene, and sesquiterpene atroctylone (Dhifi et al. 2016).

EOs contain unique aromatic natural plant sources that are often used in the cosmetic and medical industries, as well as to flavor foods and beverages (Valdivieso‐Ugarte et al. 2021; Haro‐González et al. 2021; Mehdizadeh et al. 2019; Bakry et al. 2016). The EOs of most plants are attributed as “Generally Recognized as Safe” (GRAS) (Valdivieso‐Ugarte et al. 2021; Alayoubi et al. 2020). These aromatic oily liquids are distilled from plants in various ways, like cold, dry, steam, and vacuum distillation techniques (Haro‐González et al. 2021; Massoud and Sharifan 2020; Alayoubi et al. 2020; Ahari et al. 2020; Mahmoudi et al. 2015; Burt 2004), and consist of some important compounds formed by biosynthetic pathways, such as terpenoids (the highest amount), monoterpenes, sesquiterpenes, aldehydes, aliphatic hydrocarbons, alcohols, acids, nitrogen‐containing compounds, esters (acyclic), lactones, phenols, coumarins, and sulfur‐containing compounds (Figure 1) (Alayoubi et al. 2020; Dhifi et al. 2016; Nazzaro et al. 2013; Espina et al. 2011).

**FIGURE 2:**
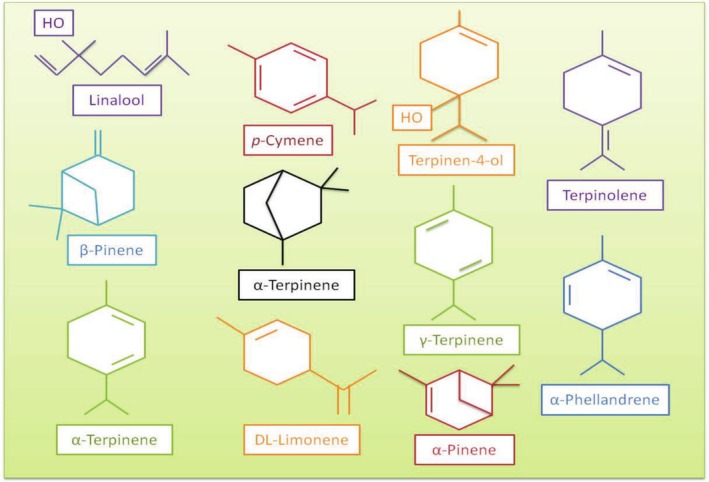
The chemical structure of some important compounds forming the complex chemical structure of EOs (Patiño‐Bayona et al. 2021).

Volatile oils exhibit antimicrobial and antibacterial activities against pathogens and spoilage bacteria in foods (Mishra et al. 2020; Alayoubi et al. 2020; Gutierrez et al. 2009, 2008). Hydrophobic properties make EOs penetrate the bacterial cell wall easily. In this way, EOs inactivate the cell after interfering with the mechanism of transportation in molecules of bacteria (Maurya et al. 2021; Rai et al. 2017). The antimicrobial effects of EOs are due to compounds found in their compositions, such as phenylpropenes (vanillin, eugenol, etc.), terpenes (e.g., limonene, *p*‐cymene), terpenoids (carvacrol, thymol, etc.), and also other compounds like isothiocyanates or alicin (Hyldgaard et al. 2012). Antimicrobial effects of volatile oils are changeable depending on the chemical structure, composition, and also functional groups of them (Vimal et al. 2013; Mohamed et al. 2013; Yesil Celiktas et al. 2007; Omidbeygi et al. 2007). EOs have a complex chemical structure (Figure 2), and among them, especially hydrophilic functional groups (e.g., hydroxyl groups in phenolic compounds, lipophilicity, etc.) provide antimicrobial properties to EOs (Patiño‐Bayona et al. 2021).

Considering other literature, antimicrobial effects in EOs are due to oxygenated terpenoids (alcohols and phenolic terpenes) and hydrocarbons. Likewise, terpenes, terpenoids, and phenolic compounds of these volatile oils are in charge of antioxidant and immunomodulatory properties, as well as antimicrobial effects (Patiño‐Bayona et al. 2021; Falleh et al. 2020; Valdivieso‐Ugarte et al. 2019; Mohamed et al. 2013).

Phenolic compounds that significantly exhibit antimicrobial effects are found especially in some plants, such as clove, oregano, rosemary, thyme, sage, and vanillin. According to other literature, it was indicated that cinnamon, clove, dill, oregano, and rosemary are the most common known plants with their impactful antimicrobial activities (Alayoubi et al. 2020; Mohamed et al. 2013; Weerakkody et al. 2010). The antimicrobial effects of EOs are variable depending upon the variety of microorganisms, and generally, Gram‐negative bacteria are more resistant than Gram‐positive bacteria against these compounds in EOs. Besides, LAB are the most stable microorganisms in comparison with Gram‐positive bacteria against EOs. These properties procure significant advantages to using EOs in probiotic products (Alayoubi et al. 2020; Mehdizadeh et al. 2019; Mahmoudi et al. 2014).

As preservatives, the EOs show an alteration depending on their resources and also composition (Falleh et al. 2020), and antimicrobial properties in EOs have been investigated in many pathogens causing various foodborne diseases (Valdivieso‐Ugarte et al. 2021, 2019). In the contamination of fermented foods, especially the EOs of citrus, dill, garlic, oregano, rosemary, and thyme, have significant antimicrobial activity against pathogens and food spoilage bacteria (Nazari et al. 2019; Mehdizadeh et al. 2019; Terpou et al. 2018; Bedoya‐Serna et al. 2018; Comunian et al. 2017; Tsiraki and Savvaidis 2016).

EOs can support the probiotics in various fermented food products. In vitro research studies showed that some plants and their EOs not only enhance the probiotics' growth but also inhibit pathogenic microorganisms. Combining EOs with probiotics may supply more antimicrobial and therapeutic characteristics, but these secondary metabolites also have an adverse effect on the viability of LAB and probiotics. Therefore, the suitable plants and their EOs should be selected for the use of probiotic products. Likewise, the selected EOs should not have negative effects on the organoleptic properties of the products containing probiotics while extending their shelf life (Keshavarzi et al. 2021; Yerlikaya 2014). The implementation of EOs alone and/or combined with probiotic bacteria in various food products is an innovative strategy to stimulate probiotics while overcoming pathogens (Mohajeri et al. 2021; Moritz et al. 2012).

In addition to their antimicrobial activities, EOs also have antioxidant effects, and these properties make EOs substantial natural components for use in the food industry, especially in extending the product shelf life (Massoud and Sharifan 2020; Ahari et al. 2020; Burt 2004).

## 3 The Effects of EOs on Microbial Interactions

**FIGURE 3:**
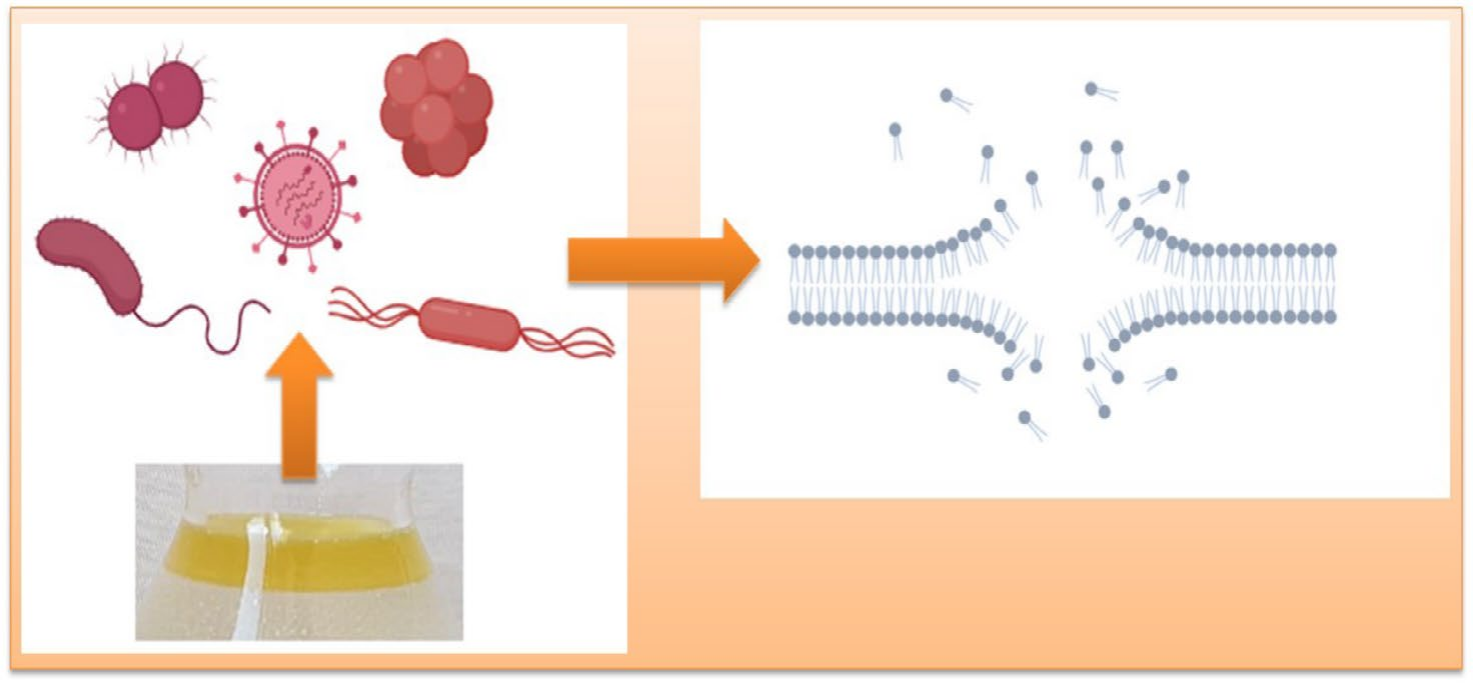
The disruption to cell membranes as a result of the application of EOs of some plants (modified from Yang et al. 2020).

In recent years, there has been an increasing focus on the exploration and development of novel antimicrobial agents derived from diverse natural sources to address the challenge of microbial resistance. Consequently, greater emphasis has been placed on the screening of antimicrobial efficacy and the refinement of evaluation methodologies. However, the antimicrobial properties of EOs obtained from different plant species vary significantly (Chouhan et al. 2017). For instance, the EOs of certain plants exert antimicrobial effects through the *disruption of the cell membrane* mechanism (Figure 3). Through this mode of action, the EO of *Cinnamomum* demonstrates activity against *E. coli* and *S. aureus* , while the EO of *Dipterocarpus gracilis* is effective against *B. cereus* and *P. mirabilis* (Zhang et al. 2016). Additionally, the *permeabilization of the membrane* mechanism is observed in the EO of *Ocimum gratissimum* , which exhibits antimicrobial activity against *P. aeruginosa* and *S. aureus* (Hyldgaard et al. 2012), whereas the EO of *Origanum vulgare* targets *S. aureus* and *P. aeruginosa* through the same pathway (Lambert et al. 2001). Moreover, the *inhibition of ergosterol biosynthesis* mechanism underlies the antifungal action of the EO of *Coriaria nepalensis* against *Candida* isolates (Ahmad et al. 2011) and the EO of *Curcuma longa* against *A. flavus* (Hu et al. 2017). In addition, the *loss of membrane integrity* mechanism is responsible for the antimicrobial effects of the EO of *Foeniculum vulgare* against *S. dysenteriae* (Diao et al. 2014), while the same mechanism is the basis of *Forsythia koreana* exhibiting activity against various foodborne and pathogenic bacteria (Yang et al. 2015). Furthermore, the EO of *Cuminum cyminum* operates through the *cytoplasmic alterations* mechanism, effectively targeting *Bacillus cereus* and *B. subtilis* , whereas the EO of *Mentha longifolia* exerts its antimicrobial action against *M. luteus* and *S. typhimurium* via the *cell wall damage* mechanism (Hyldgaard et al. 2012).

Numerous studies have been conducted to investigate the antimicrobial effects of EOs derived from various plant species. Nikolić et al. (2014) investigated the biological properties of EOs extracted from five species within the *Lamiaceae* family, namely *Lavandula angustifolia* , *Mentha piperita*, *Mentha pulegium* , *Salvia lavandulifolia*, and *Satureja montana* , focusing on their antimicrobial and cytotoxic effects as well as their chemical composition. The study examined seven clinically relevant bacterial strains, including *E. faecalis* , *L. acidophilus* , *P. aeruginosa* , *S. pyogenes* , *S. mutans* , *S. sanguis*, and *S. salivarius*, alongside 58 clinical isolates of oral *Candida* spp. and three reference strains. Among the tested EOs, *Satureja montana* exhibited the strongest antimicrobial activity, while all EOs demonstrated notable efficacy against the full spectrum of microbial species assessed in the study (Chouhan et al. 2017). Moreover, a recent investigation evaluated the antimicrobial properties of six widely utilized Brazilian culinary herbs, namely anise ( *P. anisum* L.), basil ( *O. basilicum* L.), marjoram ( *O. majorana* L.), peppermint (*M. piperita* L. var. *Piperita*), rosemary ( *R. officinalis* L.), and thyme ( *T. vulgaris* L.) against a strain of *C. perfringens* (Radaelli et al. 2016). In addition, black pepper, clove, cumin, garlic, mint, orange, oregano, thyme, and tea tree containing characteristic EOs are stated to be protective against some pathogens, such as *B. cereus* AUFE 81154, *Candida albicans* ATCC 10231, *C. oleophila* UUPP 94365, *E. coli* ATCC 25922, *Kloeckera apiculata* UUFE 10628, *L. monocytogenes* AUFE 39237, *Proteus mirabilis* AUFE 43566, *S. uvarum* UUFE 16732, *S. enteritidis* ATCC 13076, and *Metschnikowia fructicola* UUPP 23067. Besides, the EOs obtained from clove, orange, oregano, and thyme also significantly inhibit both bacteria and yeasts, whereas volatile oils of other plants like cumin, mints, and tea tree display inhibitory activities against only yeasts. Related to this, different studies have stated the antimicrobial activities of EOs (Olmedo et al. 2009, 2008).

Antibacterial effects on *Staphylococcus* and *Streptococcus* causing mastitis (Abboud et al. 2016). In the other study, the EO of perilla ( *Perilla frutescens* (L.) Britt.) exhibited strong antimicrobial activities against *L. monocytogenes* and *S. aureus* (Wang et al. 2021; Zhao et al. 2016). Moreover, Ahmad et al. (2011) reported that EO of *Coriaria nepalensis* shows antifungal activity on *Candida* isolates by damaging the integrity of the cell membrane (Ahmad et al. 2011). Besides, it was established that turmeric ( *Curcuma longa* ) has antiaflatoxigenic and antifungal effects on *Aspergillus flavus* (Hu et al. 2018). Furthermore, it was stated by Cascaes et al. (2020) that the EO of *Annona exsucca* DC has potential biological activity against pathogenic bacterial species and molecules involved in cancer development. The antimicrobial effects of *Hedychium coccineum* Buch. Ham. Ex Sm. EOs were also investigated by Arya et al. (2022). Biological components of EOs have great promise as antibacterial agents in active packaging. It was reported by Campini et al. (2021) that biodegradable Poly (lactic acid) (PLA) polymer capsules containing *Cinnamomum cassia* essential oil (CEO), eugenol (EEO), and linalool (LEO) showed colloidal stability and antibacterial activity against bacteria such as *E. coli* , *S. aureus* , *L. monocytogenes* , and *Salmonella*.

In addition to pathogens, in probiotic products, there is also a positive interaction between the EOs of some plants and probiotics. In this sense, adding some plant EOs to fermented products can give positive results to promote the viability of probiotics, while inhibiting pathogens during storage. This application is an innovative approach to improving the shelf lives of foods. In the usage of EOs in food, some parameters need to be taken into consideration, and various studies have reported that EOs can interact with carbohydrates, fat, and protein, and thus antimicrobial activity can be decreased. For example, the fat content forms a hydrophobic layer around the EOs, while phenolics interact with proteins (Mishra et al. 2020; Kostova et al. 2016; Thabet et al. 2014; Rattanachaikunsopon and Phumkhachorn 2014).

Certain EOs exhibit higher minimum inhibitory concentration (MIC) values against probiotic bacteria than against pathogenic microorganisms. This characteristic enables the simultaneous administration of probiotics and EOs for the treatment of pathogenic infections in the human gastrointestinal system (Deep et al. 2012). In probiotic dairy products, the bacteria used in their production must survive intestinal transit. The combination of plant extracts/EOs and probiotic strains enhances the intestinal modulatory effect of fermented dairy products (Azizkhani and Parsaeimehr 2018; Unusan 2020; Ozcan et al. 2021). Various studies have extensively examined yogurt enriched with EOs to prolong its shelf life, leveraging the intrinsic antibacterial, antifungal, and antioxidant properties of EOs and highlighting their potential as natural preservatives (Masyita et al. 2022). Advances in biopolymer‐based nanoemulsions incorporating EOs have facilitated improvements in oxidative stability, thermal resistance, shelf life, and additional bioactive properties in food products, thereby reducing reliance on synthetic preservatives (Rehman et al. 2021). A notable example is the incorporation of *Melissa officinalis* EO into microcapsules, which demonstrated an enhancement in the antioxidant activity of yogurt samples (Sani et al. 2020).

The ethanolic extract and EO of *Ferulago angulata* were incorporated into probiotic yogurt containing *Lactobacillus acidophilus* and *Bifidobacterium bifidum* , and their impact on probiotic bacterial viability during storage was assessed. The survival rate of both probiotic strains in yogurt fortified with *Ferulago angulata* EO (0.03%) was significantly higher than in the control group, yielding favorable results in protein profiling, physicochemical properties, microbial stability, and sensory evaluation (Keshavarzi et al. 2021). Additionally, the viability of probiotics, antioxidant activity, and sensory acceptability of yogurt enriched with the EOs of *zataria*, basil, and peppermint were examined to enhance its antioxidant potential. Among these, yogurt samples containing peppermint and basil EOs exhibited both strong antiradical activity and high sensory acceptability (Azizkhani and Parsaeimehr 2018).

Furthermore, the EOs of eucalyptus ( *Eucalyptus camaldulensis* ) and myrrh (*Commiphora myrrha*) were incorporated into fortified buffalo set yogurt to evaluate their potential health benefits. Their effects on sensory attributes, texture, antibacterial activity, total phenolic content, and antioxidant capacity were analyzed. Among the formulations, yogurt enriched with eucalyptus oil (0.9%) exhibited the highest antioxidant activity and total phenolic content. The findings suggest that both eucalyptus and myrrh EOs can be effectively utilized in yogurt production to enhance its physicochemical properties and promote health benefits (Hamed et al. 2021).

Other probiotic‐rich fermented foods include beverages such as fermented milk. In a separate study, three probiotic curd‐based beverages were formulated with varying concentrations of EOs derived from *Coleus aromaticus*, *Rama tulasi*, and *Shyama tulasi*. These beverages were subsequently co‐cultured with common enteric pathogens in equal concentrations, and microbial growth was quantified. The results demonstrated that the probiotic beverages effectively inhibited pathogen proliferation, and their shelf life was significantly extended compared to conventional probiotic formulations. These findings suggest that such beverages possess considerable potential in preventing enteric infections. Collectively, these studies highlight the significant antimicrobial properties of both probiotics and EOs, emphasizing their combined application as a promising strategy for the development of functional foods with enhanced health benefits (Deep et al. 2012; Jaiswal et al. 2009).

In a study, it was revealed that EOs of *Mentha spicata* and *Mentha aquatic* have antibacterial effects against *Bifidobacterium animalis* , *Clostridium perfringens* , *Lactobacillus reuteri* , and *S. aureus* in kashk (fermented drained milk product) (Massoud and Sharifan 2020; Ahari et al. 2020; Golestan et al. 2016). These effects make the EOs appropriate for use as bio‐preservative ingredients in functional foods (Valdivieso‐Ugarte et al. 2021).

**FIGURE 4:**
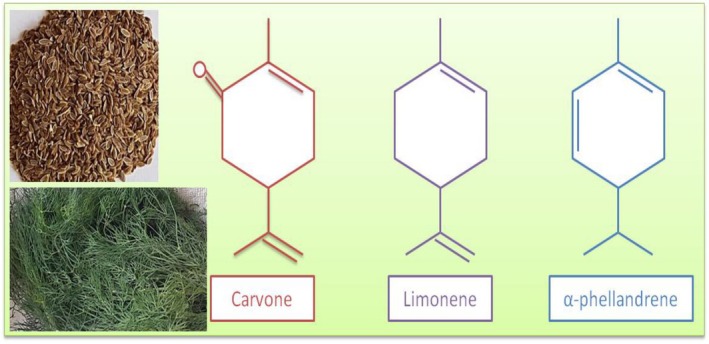
The chemical structures of carvone, limonene, and α‐phellandrene (Gajić et al. 2020).

**FIGURE 5:**
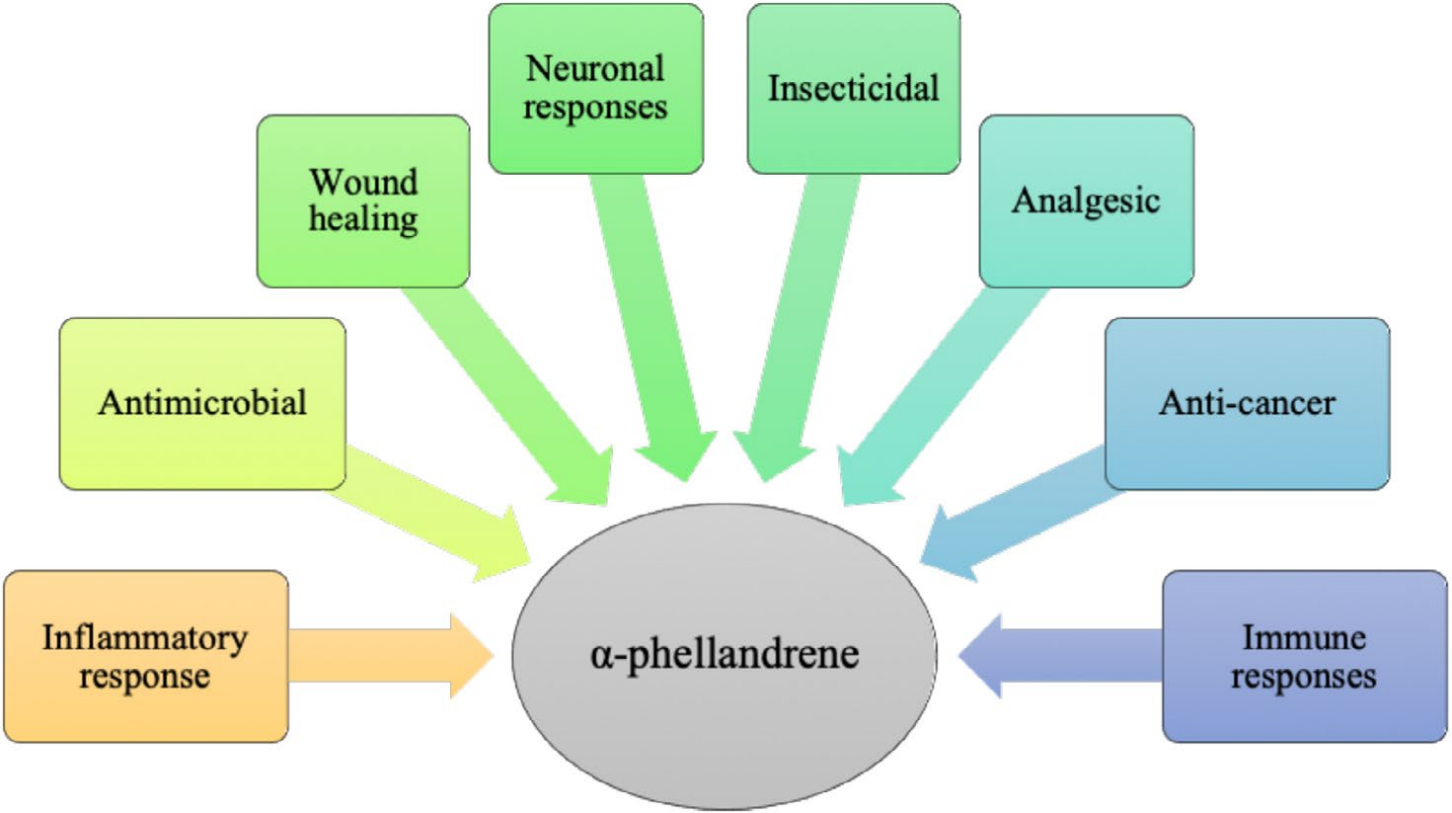
The biological activities of α‐phellandrene (Thangaleela et al. 2022).

Dill ( *Anethum graveolens* ), a member of Apiaceae (Umbelliferae), is an aromatic herbaceous plant. Main compounds of essential oil in dill (DEO) are carvone, limonene, and α‐phellandrene (Figure 4) (Gajić et al. 2020; Mehdizadeh et al. 2019; Keskin and Baydar 2016; Ramadan et al. 2013; Babri et al. 2012; Radulescu et al. 2010). These compounds (e.g., biological properties of α‐phellandrene shown in Figure 5) have substantial biological activities, and thus dill exhibits some important biological effects due to its EO, including antibacterial, antimycobacterial, and antioxidant properties, etc., and especially these properties provide DEO to use in the food industry to enhance the shelf life of products and also to exhibit antimicrobial activity against pathogens (Milenković et al. 2024; Masoody et al. 2023; Mohamed et al. 2013). In a study, DEO was combined with *L. casei* and *B. bifidum* at concentrations of 50 ppm and 100 ppm in yogurt. As a result, the viability of these probiotics improved after 2 weeks and afterward declined after 21 days of storage. Likewise, the 100 ppm of DEO incorporation provided an acceptable level of probiotics (useful healthy application). On the other hand, the 100 ppm of DEO provided both enhancing the shelf life with admissible probiotic numbers and procuring appropriate color and texture without growing and also any indications of spoilage microorganisms. Consequently, DEO is both safe and nontoxic for consumers' health; hence, these secondary metabolites can be used as natural components instead of synthetic additives (Mehdizadeh et al. 2019).

In the other study, the yogurt was fortified with DEO, and the data indicated that the growth of *L. delbrueckii* subsp. *bulgaricus* and *S. thermophilus* was not affected by DEO. It was observed that pathogens ( *L. monocytogenes* and *E. coli* O157:H7) in the products were inhibited, and the more resistant pathogen was *E. coli* O157:H7 compared to *L. monocytogenes* . Besides, Gram (+) bacteria were observed to be more sensitive than Gram (−) bacteria. Thus, it was recommended that DEO could be applied to foods successfully as a natural metabolite in yogurt (Mohamed et al. 2013).

**FIGURE 6:**
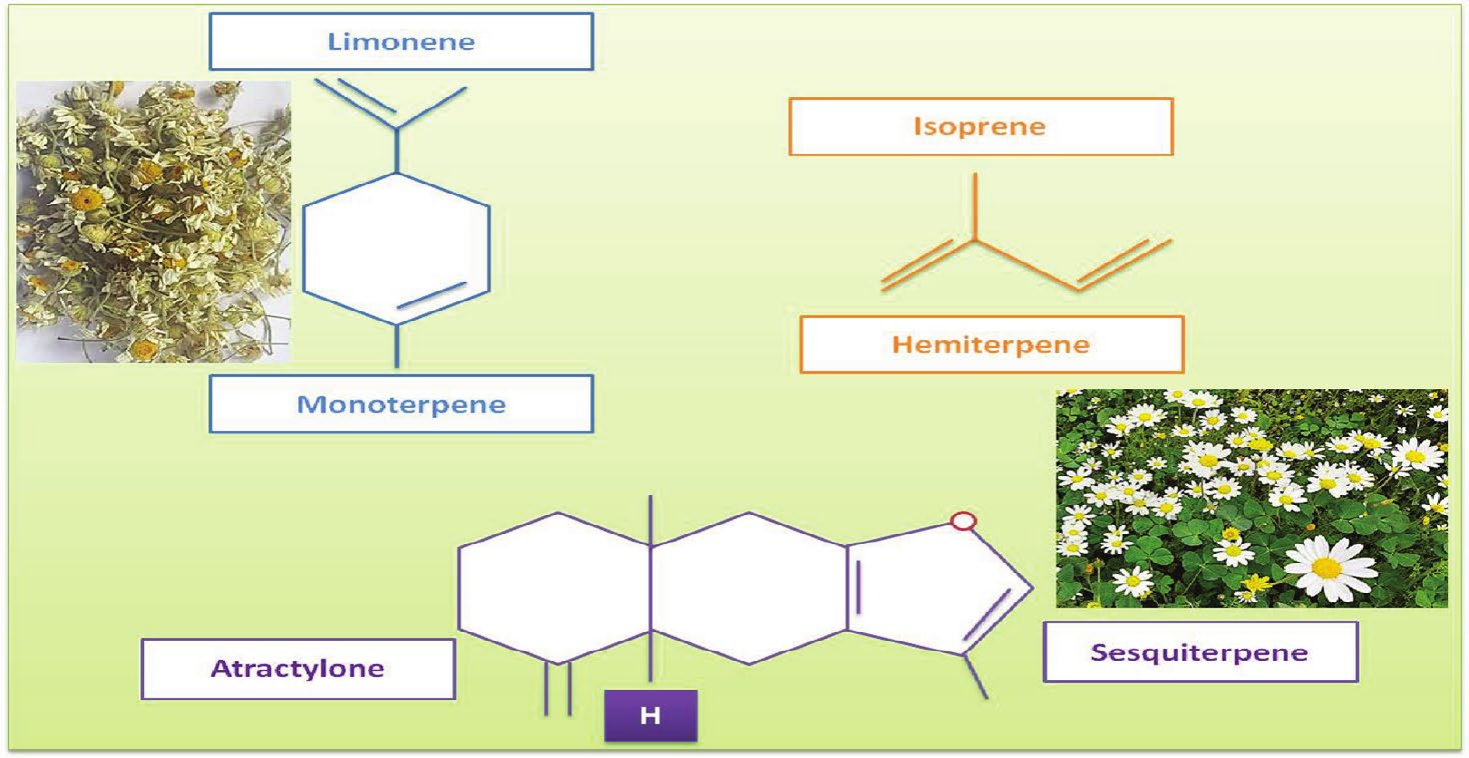
The chemical structure of (E)‐β‐farnesene, germacrene D, and α‐bisabolol oxide A (Al‐Ghanim et al. 2023).

Chamomile ( *Matricaria recutita* ), belonging to Asteraceae, is a fragrant plant with white and yellow flowers (Jeshni et al. 2015). The main compounds in the essential oil of chamomile (CEO) are (E)‐β‐farnesene, germacrene D, and α‐bisabolol oxide A (Figure 6). Chamomile displays some important biological activities, such as anti‐inflammatory, antiseptic, carminative, sedative, etc., due to its CEO (Al‐Ghanim et al. 2023; Salem et al. 2019; Heidari and Sarani 2012; Baghalian et al. 2011; Ogata et al. 2010). In this regard, the CEO can be used in many industries (Massoud and Sharifan 2020). In a study, CEO was applied to yogurt containing probiotic at the rate of 0.2% and 0.4%, and probiotic *B. lactis* BB‐12 remained the largest number with the concentration of 0.4% CEO (Massoud and Sharifan 2020; Ahari et al. 2020; Yangilar and Yildiz 2018). Moreover, in the other study, it was revealed that chamomile, ginger, and the combination of EOs of these plants are rather influential in controlling spoilage microbial growth. In conclusion, the CEO can be used to enhance the properties of yogurt containing *B. lactis* BB‐12 (Yangilar and Yildiz 2018).

**FIGURE 7:**
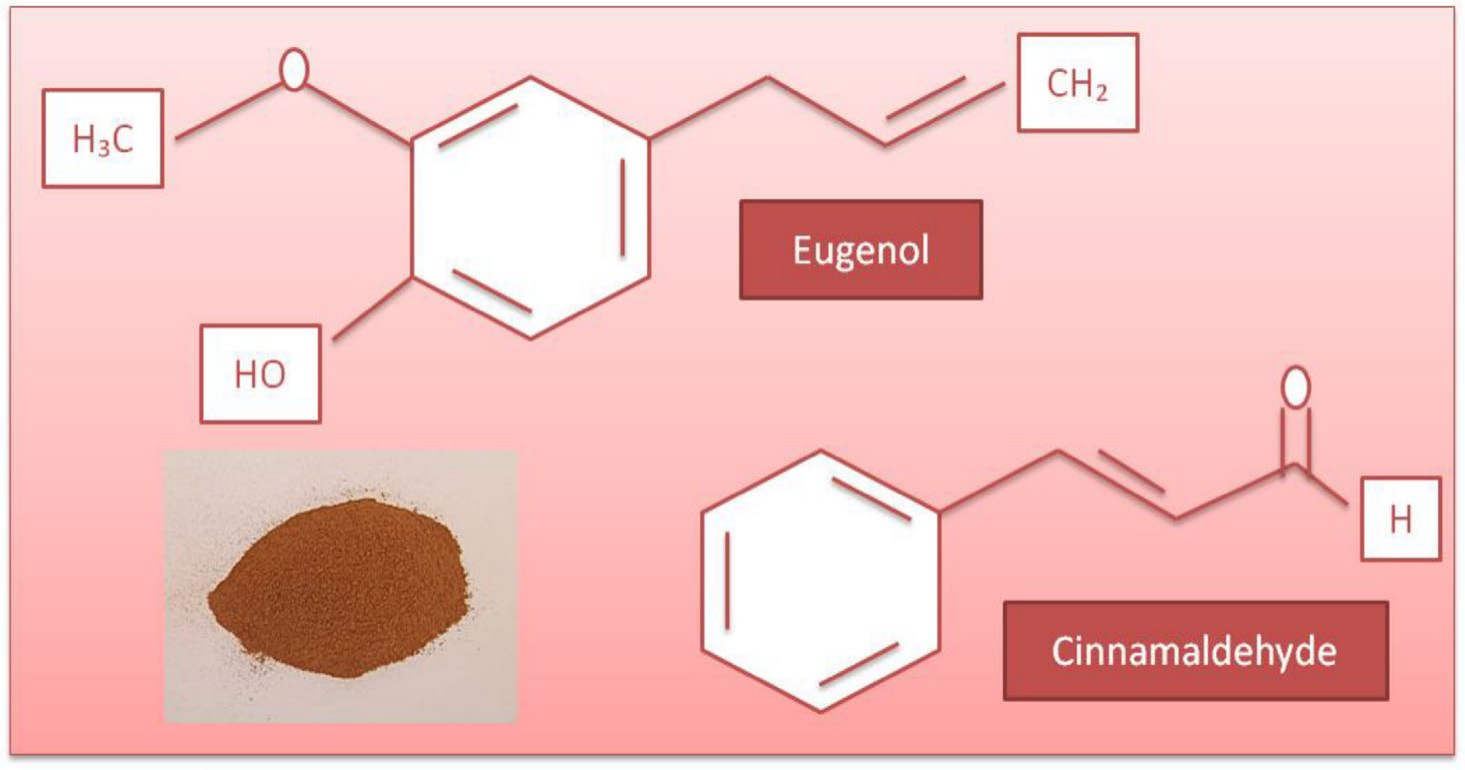
The chemical structures of eugenol and cinnamaldehyde (Feltes et al. 2023).

Cinnamon ( *Cinnamomum verum* ), a member of Lauraceae, is an aromatic plant commonly used as a spice, especially in desserts, sweets, etc. The characteristic smell of cinnamon is also due to the EO it contains (Wijesinghe, de Feiria, et al. 2020; Petrović et al. 2010). The main compounds in the essential oil of cinnamon (CIEO) are cinnamaldehyde and eugenol (Figure 7) (Feltes et al. 2023; Wijesinghe, Maia, et al. 2020; Paranagama et al. 2010; Singh et al. 2007), and compounds of CIEO change depending on aerial parts (fruit, leaf, and bark) of the plant, such as cinnamyl acetate and coumarin in fruit; eucalyptol and cinnamaldehyde in leaf; cinnamaldehyde, linalool, caffeic acid, benzoic acid, trans‐cinnamaldehyde, and camphor in bark (Błaszczyk et al. 2021). Cinnamaldehyde and eugenol are the most bioactive compounds of EO (Cho et al. 2020; Mehdizadeh et al. 2019), and CIEO exhibits antimicrobial effects significantly on saprophytes and pathogens owing to its bioactive components. In a study, CIEO was applied to products, and according to MIC results by using resazurin, no microbial growth was monitored. The MIC value for CIEO was found below 0.025% v/v. Nevertheless, as regards the reports of many studies, the EOs must be applied at a ratio higher than their in vitro MICs for displaying antimicrobial activities in food matrix (Valdivieso‐Ugarte et al. 2021; Denkova‐Kostova et al. 2020; Moritz et al. 2012).

However, CIEO was found to be close to the in vitro MIC value of 0.04%. So, CIEO had a great effects on starter culture in the food system. Although the volatile oils of this plant influenced the starter culture significantly in yogurt, *Lactobacillus rhamnosus* was not affected considerably (only reduced by 1 log), and the count of this probiotic bacterium was above 10^6^ CFU/mL, necessary for a product to be considered a probiotic. Consequently, the fermentation of milk products with starter cultures is impossible by using CIEO except for *L. rhamnosus* , and probiotic dairy products can be produced using both *L. rhamnosus* and CIEO (Moritz et al. 2012).

In the other study, the effects of CIEO at the different concentrations (0.1%, 0.01%, and 0.001%) on probiotic bacteria ( *L. paracasei* CNCM I‐4034, *B. breve* CNCM I‐4035, and *L. rhamnosus* CNCM I‐4036) and pathogens ( *E. coli* CECT 729, *E. coli* CECT 501, *S. typhi* CECT 725, and *S. typhimurium* CECT4594) were investigated. According to results, CIEO exhibited antimicrobial effects on pathogens (especially *E. coli* CECT 729 and *S. thyphi* CECT 725) significantly and inhibited the probiotic strains at 0.1% (v/v). This effect was correlated to the occurrence of cinnamic aldehyde and also eugenol (Swamy et al. 2016). But, it is observed that CIEO concentration between 0.01 and 0.001% has no inhibitory influence on probiotic strains. However, the rate of CIEO at 0.01% also inhibited *Bacteroides* spp. and *Trichococcus pasteurii* considerably (Valdivieso‐Ugarte et al. 2021).

Moreover, Friedman (2017) indicated that cinnamaldehyde prevents foodborne diseases exhibiting inhibitory activities against *B. cereus* , *Campylobacter jejuni* , *Clostridium perfringens* , *E. coli* , *L. monocytogenes* , and *S. enterica* in cheese, etc. (Friedman 2017). Furthermore, in the other study, both the EO of cinnamon and the lactoperoxidase system displayed synergistic inhibitory effects on *Salmonella* in milk flora for 5 days at 4°C (Abbes et al. 2018).

**FIGURE 8:**
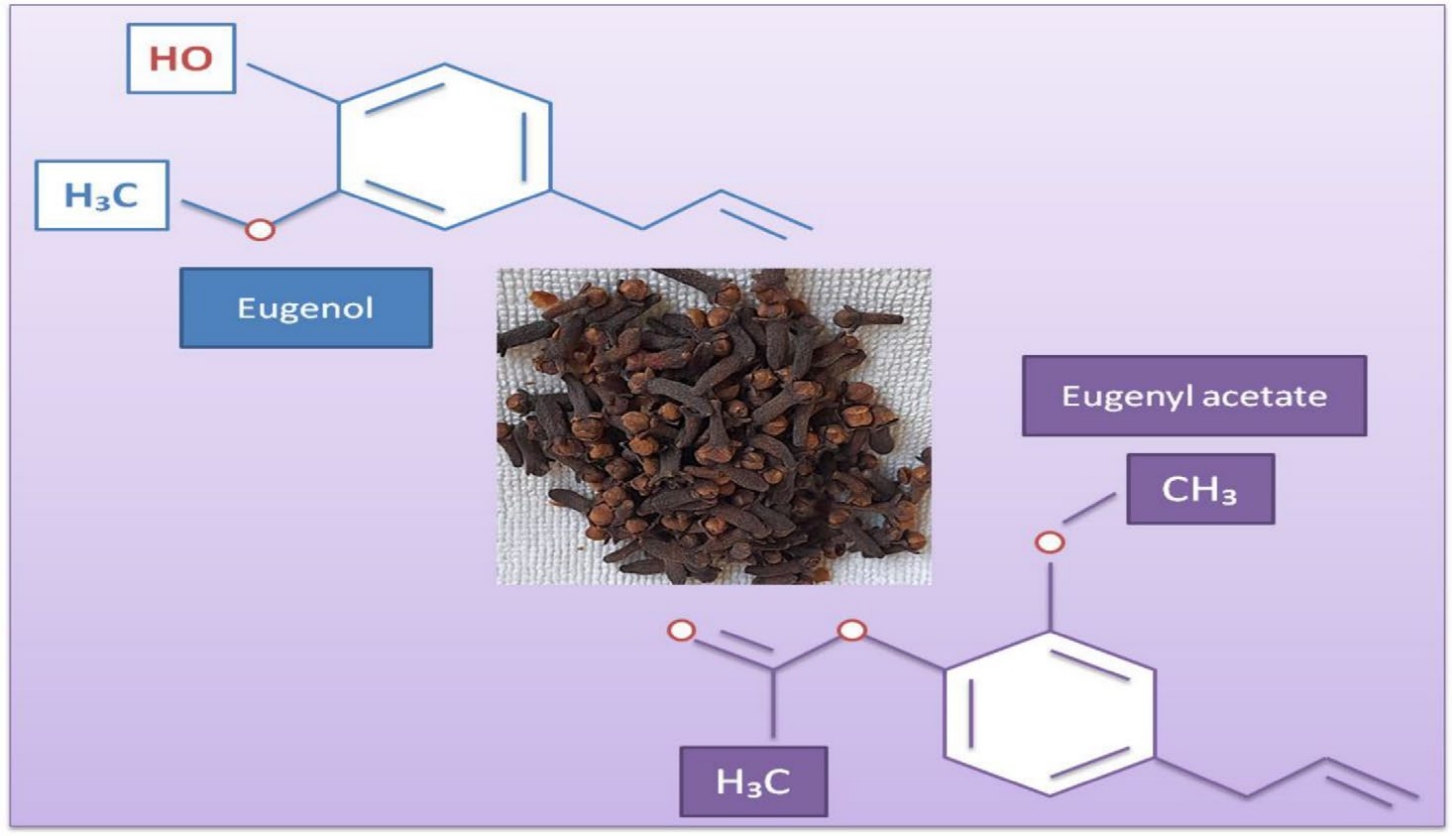
Dried form of *Syzygium aromaticum* L. samples (Haro‐González et al. 2021).

Clove ( *Syzygium aromaticum* L.), belonging to Caryophyllaceae, is a characteristic, intensely aromatic and odorous plant containing essential oil (CLEO), and dominant compounds in CLEO consist of eugenol and eugenyl acetate (Figure 8), as well as β‐caryophyllene and α‐humulene. CLEO, due to its compounds (eugenol, α‐humulene, eugenyl acetate, β‐caryophyllene, etc.), owns antimicrobial, antioxidant, anti‐inflammatory, antiviral, analgesic, anticancer, insecticidal, etc. activities and effects, and CLEO can be used widely in some applications, such as flavoring, food, cosmetic, and perfumery industries, etc. (Haro‐González et al. 2021; Alayoubi et al. 2020).

In a study, CLEO was applied to products, and in reference to MIC results by using resazurin, no microbial growth was observed. The MIC value for CLEO was revealed to be 0.2% v/v. The maximum acceptance of the concentration organoleptically in yogurt was lower than the MIC values obtained for CLEO. Besides, it was observed that CLEO did not have a fatal effect on both *L. rhamnosus* and starter cultures. Therefore, the use of CLEO does not reduce the fermentation. As a consequence, both *L. rhamnosus* and starter cultures demonstrate resistance to CLEO, and this volatile oil can be used in probiotic products (Moritz et al. 2012). In the other study, the effects of CLEO at the different concentrations (0.1%, 0.01%, and 0.001%) on probiotic bacteria ( *L. paracasei* CNCM I‐4034, *B. breve* CNCM I‐4035, and *L. rhamnosus* CNCM I‐4036) and pathogens ( *E. coli* CECT 729, *E. coli* CECT 501, *S. typhi* CECT 725, and *S. typhimurium* CECT4594) were investigated. According to the results, CLEO exhibited antimicrobial effects on pathogens (especially *E. coli* CECT 729 and *S. thyphi* CECT 725) significantly, and inhibited the probiotic strains at 0.1% (v/v). This effect was correlated with the occurrence of eugenol (Swamy et al. 2016). But, it is indicated that CLEO concentration between 0.01% and 0.001% has no inhibitory influence on probiotic strains (Valdivieso‐Ugarte et al. 2021).

**FIGURE 9:**
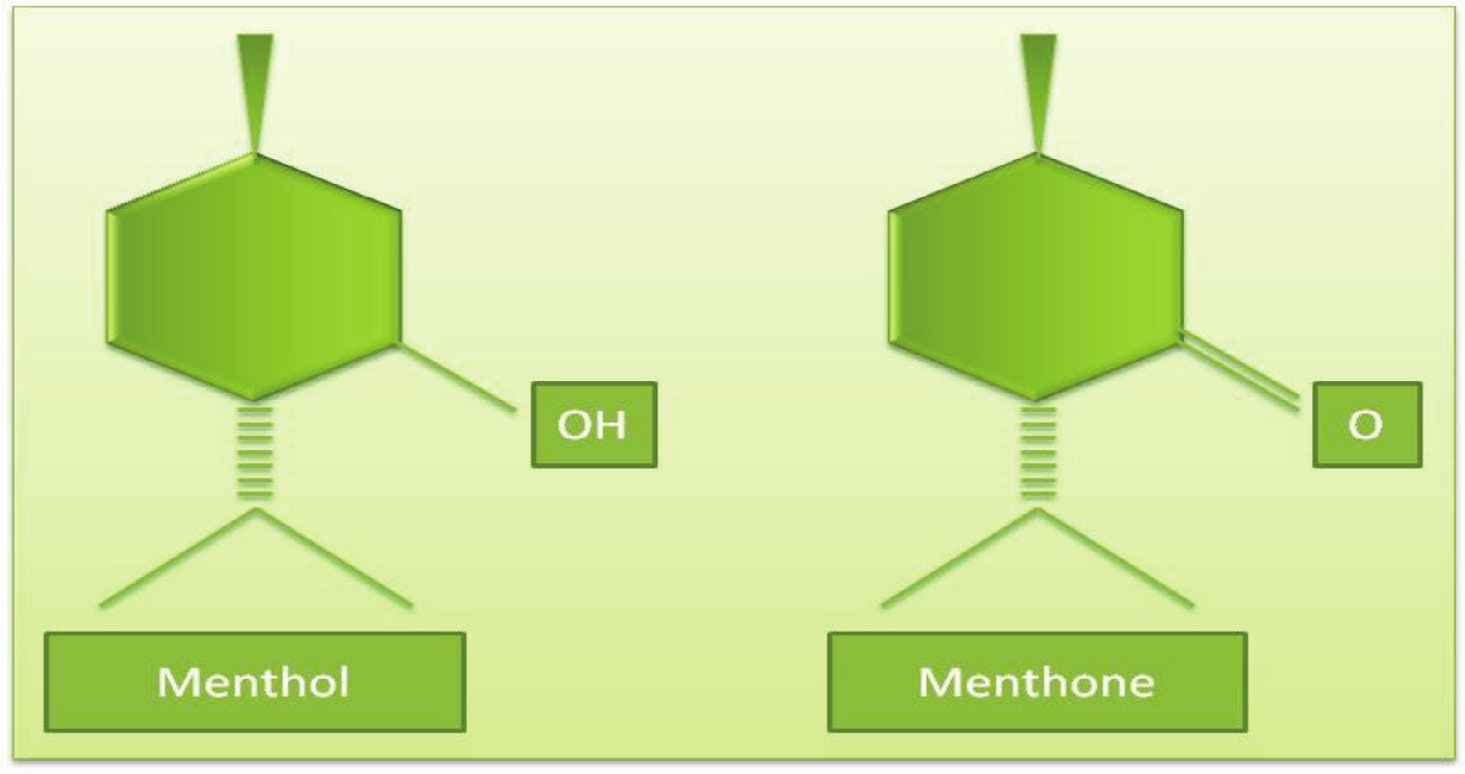
The chemical structures of menthol and menthone (Samber et al. 2015).

Peppermint (*Mentha piperita* L.), a member of Labiatae, is an intense aromatic plant rich in EO that was obtained by crossing *M. spicata* and *M. aquatica* . The chief compounds in the EO of peppermint (PMEO) consist of menthol and menthone (Figure 9) (Patil et al. 2016; Samber et al. 2015; Johari et al. 2015; Figueroa‐Pérez et al. 2015; Mahboubi and Kazempour 2014). The major constituents of PMEO are menthol, menthone, and menthyl acetate, medicinally significant compounds representing approximately 70% of total EOs (Azizkhani and Tooryan 2016; Schmidt et al. 2009). These compounds enable peppermint to be used widely in many applications (Moritz et al. 2012; Derwich et al. 2010). PMEO is especially important for food products due to its moderate antimicrobial activities on both Gram (+) and Gram (−) bacteria (Azizkhani and Tooryan 2016; Singh et al. 2015).

In a study, PMEO was used in dairy products, and considering MIC results by using resazurin, microbial growth was not monitored. The MIC value for PMEO was found to be 0.4% v/v. The maximum acceptance of the concentration organoleptically in yogurt was lower than the MIC values obtained for PMEO. Besides, it was observed that PMEO did not have a bacteriostatic effect on both *L. rhamnosus* and starter cultures. Therefore, the use of PMEO does not reduce the fermentation. In conclusion, both *L. rhamnosus* and starter cultures exhibit resistance to MEO, and this secondary metabolite can be applied in probiotic products (Moritz et al. 2012). In another study, firstly, the production of probiotic yogurt was provided by fortifying it with probiotic bacteria ( *L. acidophilus* LA5, *L. fermentum* , and *B. bifidum* BB‐12), starter cultures, and also PMEO to the product, and some substantial factors such as antimicrobial activity, pH value, survivability of probiotic bacteria, and counts of the final product were evaluated at storage for 0, 7, 14, and 28 days at a temperature of 4°C, respectively. In regard to the results, it was observed that yogurt treated with PMEO inhibited *L. monocytogenes* more than *E. coli* due to its main phenolic compounds during 28 days of storage. On the other hand, the final probiotic yogurt obtained by adding the mentioned probiotic bacteria was produced successfully with an acceptable viable count of probiotics. Consequently, the probiotic yogurt can be produced with PMEO to improve the potential functionality of the final product (Azizkhani and Tooryan 2016).

**FIGURE 10:**
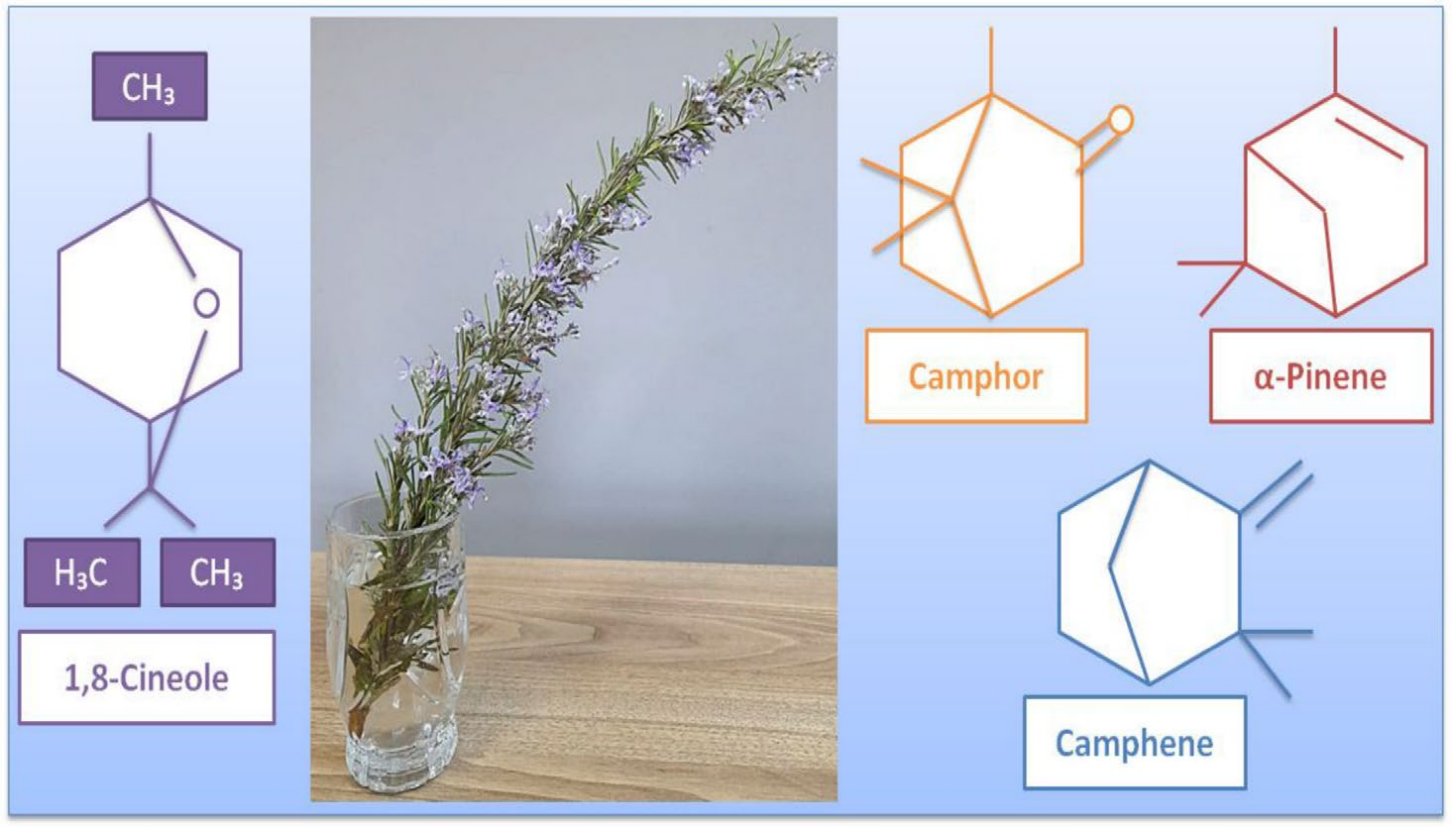
The main components of EO in rosemary ( *Rosmarinus officinalis* ) (Ouknin et al. 2021).

Rosemary ( *Rosmarinus officinalis* ), belonging to Lamiaceae, is an aromatic herbaceous plant (Di Cesare Mannelli et al. 2016; Abadi et al. 2016) that can be used in many applications (e.g., taste, flavor, preservation of foods, etc.) and has biological properties (suppressing gluconeogenesis, down‐regulation of glycogen synthesis, inducing glycolysis, increased cell glucose consumption, lowering blood cholesterol level, increased insulin sensitivity, etc.) due to its EO. Rosemary is an important plant, especially due to the antimicrobial and antioxidant effects of CEO, and these properties allow it to be used in food preservation, enhancing shelf life, inhibiting pathogens, etc. (Alayoubi et al. 2020; Medicherla et al. 2016; Habtemariam 2016; Nazem et al. 2015). The major compounds in the essential oil of rosemary (REO) are camphor, 1,8‐cineole, camphene, and α‐pinene (Figure 10) with antimicrobial activities (Shiravi et al. 2021; Aouadi et al. 2021; Massoud and Sharifan 2020; González‐Minero et al. 2020; Ahari et al. 2020; Andrade et al. 2018; Satyal et al. 2017). In addition, the average phenol content in total REO was 8.93 ± 0.11 mg gallic acid/g (Massoud and Sharifan 2020).

In a study, the probiotic yogurt was produced by fortifying starter culture ( *L. delbrueckii* subsp. *bulgaricus* and *S. thermophilus* ) with probiotic ( *B. bifidum* ). Besides, REO was applied additionally at the rates of 1%, 2%, and 3% (Massoud and Sharifan 2020). According to data, REO displayed a remarkable potential to maintain adequately the population of both probiotic *B. bifidum* and also the starter cultures in yogurt. In addition to this, the sample with 1% REO provided maximum conditions on flavor, odor, viscosity, and texture as well as overall acceptability during storage. On the other hand, results showed that both biological, physicochemical, and rheological properties and sensory scores of probiotic yogurts were enhanced by the higher level of phenolic compounds found in REO (Massoud and Sharifan 2020). Moreover, the EOs of rosemary and oregano were used together in Minas Frescal cheese, and it was investigated the interaction between probiotics and pathogens. According to results, both EOs of these plants had synergistic effects in retarding the growth of *L. acidophilus* LA‐5, while not decreasing the numbers of this bacterium at cold storage. Besides, terpenes in the composition of both EOs decreased the number of the growing of *E. coli* O157: H7 during storage for 21 days (Diniz‐Silva et al. 2020).

**FIGURE 11:**
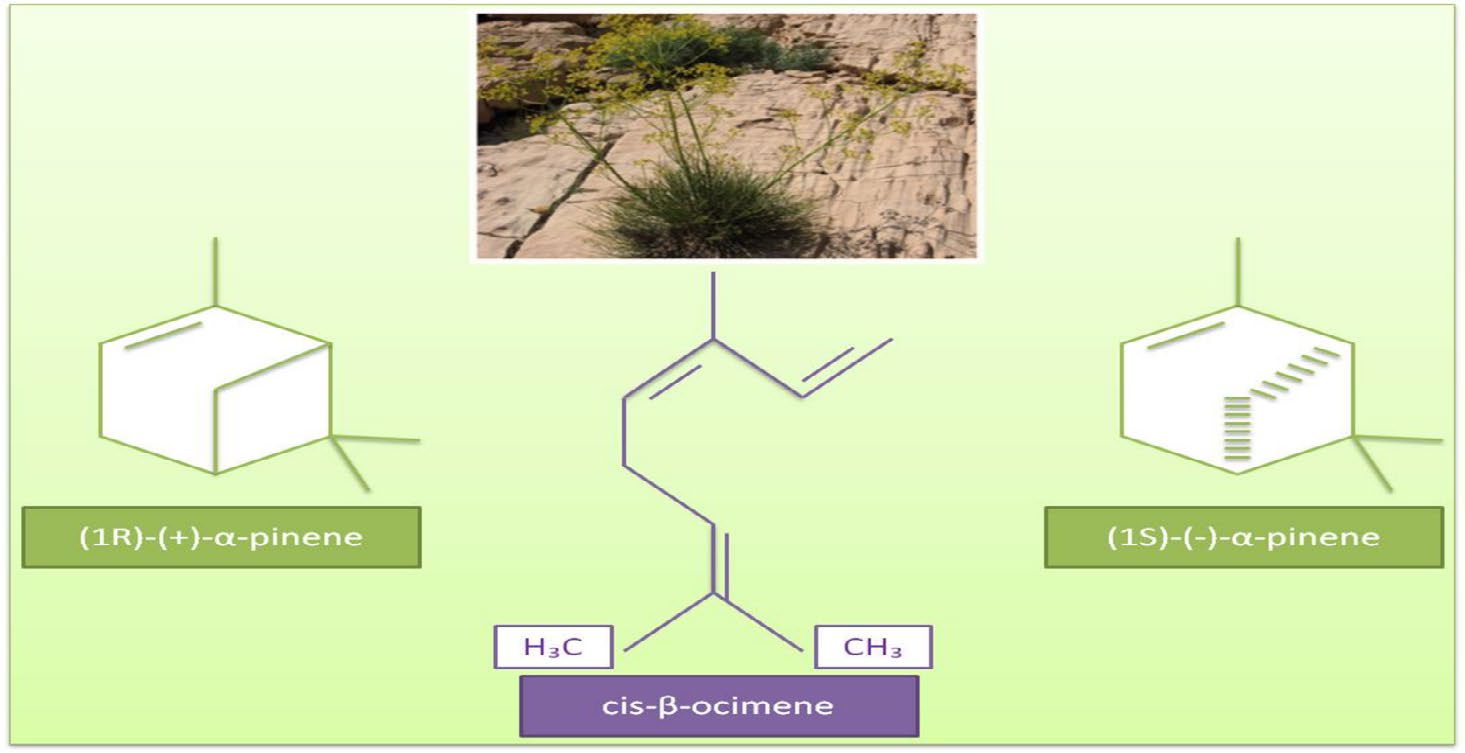
The chemical structures of α‐pinene and cis‐β‐ocimene in *Ferulago angulata* Boiss. (Salehi et al. 2019; Lorigooini et al. 2019; Farré‐Armengol et al. 2017).

Chavir (*Ferulago angulata* Boiss.), pertaining to Apiaceae, is an aromatic plant, native to Iran, and has antibacterial, antioxidant, antiparasitic, and sedative effects. It can be used in food products to preserve and enhance the shelf life (Alizadeh et al. 2019; Ghasemi Pirbalouti et al. 2016; Azarbani et al. 2014). Besides, the aerial section of chavir is added to foods as a flavoring. *Ferulago angulata* contains EO, and the major compounds in the essential oil of chavir (CHEO) comprise α‐pinene and cis‐β‐ocimene (Figure 11) (Salehi et al. 2019; Farré‐Armengol et al. 2017; Ghasemi Pirbalouti et al. 2016).

In a study, yogurt was produced with starter cultures ( *L. delbrueckii* ssp. *bulgaricus* and *S. thermophilus* ) and probiotics ( *L. acidophilus* LA‐5 and *B. bifidum* BB‐12). Likewise, CHEO was added at the concentrations of 0.01% and 0.03%. About the results, it was observed that *B. subtilis* , *E. coli* , *L. delbrueckii* subsp. *bulgaricus, S. aureus*, and *S. thermophilus* were inhibited, while *L. acidophilus* and *B. bifidum* were viable and alive. Besides, the counts of molds and yeasts were reduced with CHEO (*p* < 0.01). On the other hand, the products should be consumed until the 14th day with enough amounts of probiotic, and also CHEO fortified at the rate of 0.03% is the best implementation due to ensuring desired physicochemical and sensory properties (Keshavarzi et al. 2021).

**FIGURE 12:**
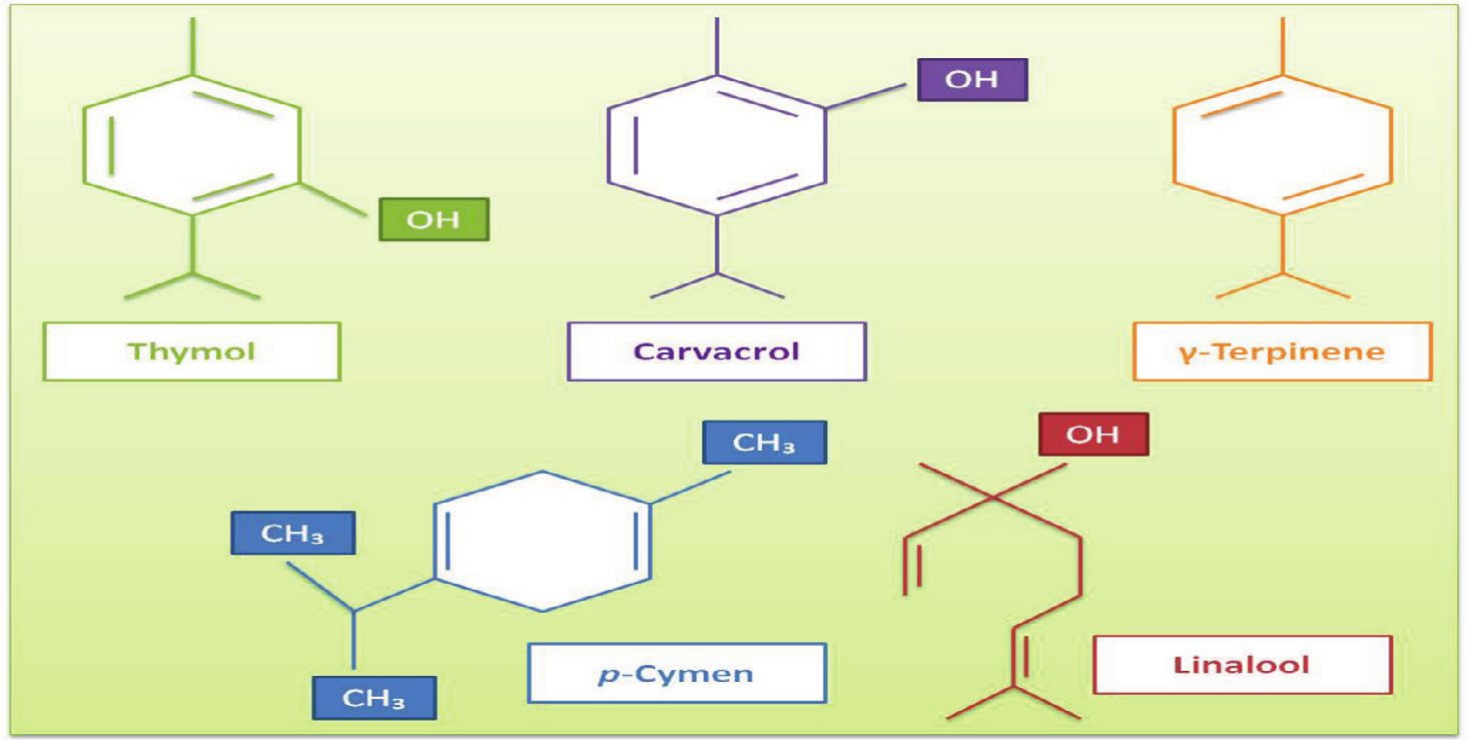
The main components of EO in *Zataria* (Vassiliou et al. 2023; Ghorani et al. 2022).

*Zataria* ( *Z. multiflora* Boiss.) commonly known as “Sattar” or “Zattar”, regarding Labiatae, is an aromatic and medicinal plant (Morvaridi et al. 2025; Bakhtiar et al. 2025; Saei‐Dehkordi et al. 2010). The aromatic properties of *Zataria* are due to its EO, and the dominant components in the volatile oil of *Zataria* (ZEO) are carvacrol, γ‐terpinene, and α‐pinene, and according to other literature, the main compounds of ZEO consist of thymol (41.8%), carvacrol (28.8%), and *p*‐cymene (8.4%). The primary compounds are shown in Figure 12. (Vassiliou et al. 2023; Ghorani et al. 2022; Azizkhani and Tooryan 2016).

**FIGURE 13:**
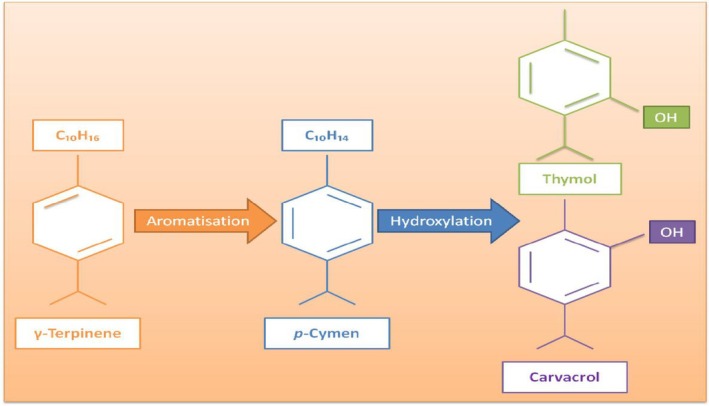
The formation pathway of thymol and carvacrol (Taherian et al. 2009).

Among these compounds, thymol and carvacrol are biosynthesized by γ‐terpinene and *p*‐cymene (Figure 13) (Taherian et al. 2009). These compounds have antimicrobial effects, and this characteristic ensures ZEO is used in the food industry for enhancing shelf life, inhibiting pathogens, etc. In a study, probiotic yogurt was produced by fortifying it with probiotic bacteria ( *L. acidophilus* LA5, *L. fermentum* , and *B. bifidum* BB‐12), starter cultures, and ZEO. Afterwards, some important parameters (e.g., antimicrobial activity, pH value, viable probiotic counts, etc.) for the final product were evaluated at 0, 7, 14, and 28 days of storage at 4°C, respectively. Considering the results, ZEO‐treated yogurt displayed the greatest inhibitory effect on *E. coli* and *L. monocytogenes* owing to its main phenolic compounds during 28 days of storage. Besides, it was indicated that *L. monocytogenes* is more sensitive than *E. coli* to the inhibiting activity of ZEO. On the other hand, the yogurt obtained with the fortification of the mentioned probiotic bacteria were produced successfully with an acceptable viable count of probiotics. In conclusion, probiotic yogurt can be produced with ZEO due to its improvement on the potential functionality of the final product (Azizkhani and Tooryan 2016).

Basil ( *Ocimum basilicum* ), a member of Lamiaceae, is an aromatic plant widely cultivated and used in some industries, such as cosmetics, food, medicine, perfumery, pesticides, etc. The aromatic characteristic of this plant results from some compounds of its EO (especially 1,8‐cineole, methyl cinnamate, methyl chavicol, and linalool), and the main compounds in the EO of basil (BEO) are linalool, chavicol, (Z)‐α‐bergamotene, and 1,8‐cineole (Ciriello et al. 2025; Zhakipbekov et al. 2024; Radulovic et al. 2013; Govindarajan et al. 2013). In addition to its aroma, the major compounds of BEO provide the plant with some significant biological properties like antimicrobial activity on Gram (−) and Gram (+) bacteria, yeasts, and also molds. This property is crucial to the application of BEO in food products and their shelf life. In a study, firstly, probiotic yogurt was produced with the addition of probiotic bacteria ( *L. acidophilus* LA5, *L. fermentum* , and *B. bifudum* BB‐12), starter cultures, and BEO, and then, antimicrobial activity, pH value, and viability of probiotic counts of the final product were evaluated at 0, 7, 14, and 28 days of storage at 4°C, respectively. In reference to data, yogurt applied BEO exhibited a significant inhibitory effect on *E. coli* and *L. monocytogenes* owing to its basic phenolic compounds during 28 days of storage. Besides, *L. monocytogenes* was revealed to be more sensitive than *E. coli* against the inhibiting activity of BEO. On the other hand, the yogurt obtained by adding these probiotic bacteria was produced successfully with acceptance in counts of living cells (Ciriello et al. 2025; Azizkhani and Tooryan 2016; Nguyen and Niemeyer 2008).

**FIGURE 14:**
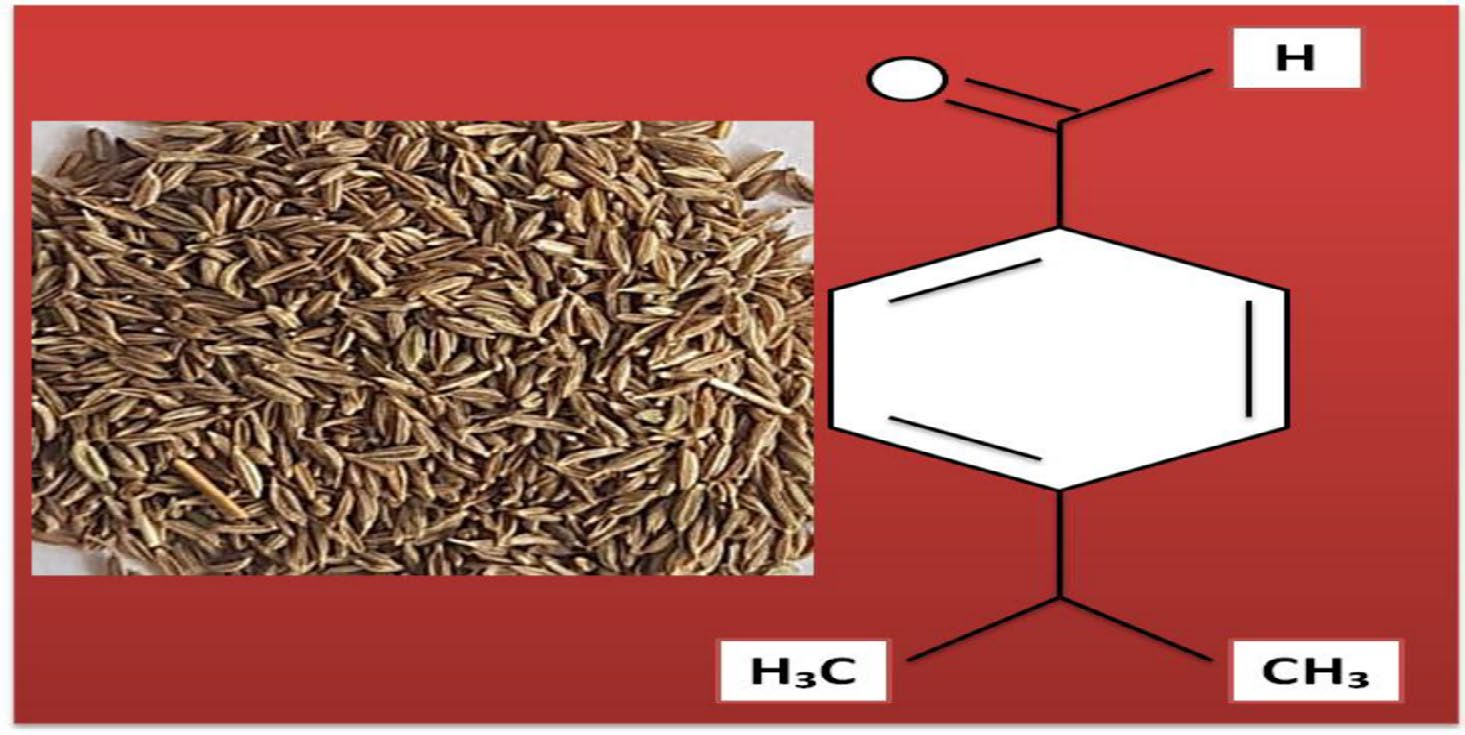
The chemical structure of cuminaldehyde (Ebada 2017).

Cumin ( *Cuminum cyminum* ), belonging to Apiaceae, is an aromatic plant containing volatile oil (Boughendjioua 2019; Bose 2018; Mehdizadeh et al. 2017; Wei et al. 2015). The major constituent in the volatile oil of cumin (CUEO) is cuminaldehyde, a fundamentally dominant smelling component of CUEO (Figure 14) (Ahari et al. 2020; Singh et al. 2017). Likewise, the other main compounds in CUEO comprise γ‐terpinene, limonene, and α‐ and β‐pinene (Ahari et al. 2020; Mehdizadeh et al. 2017). Cumin aldehyde, γ‐terpinene, and β‐pinene are bioactive components in CUEO, and especially cumin aldehyde exhibits antibacterial and antifungal activities. Besides, the antibacterial effects of CUEO can change depending upon the composition and concentration of nutrition, the naturality of metabolites in organisms, and the temperature of storage (Mohajeri et al. 2021; Singh et al. 2017; Mahmoudi 2013). In a study, yogurt was produced with *L. delbrueckii* subsp. *bulgaricus, S. thermophilus*, and *B. bifidum* . Then, CUEO was added at the rates of 1%, 2%, and 3%. According to the results, CUEO exhibited a remarkable ability to deliver *B. bifidum* with an adequate population, but decreased the growth concentration of starter cultures (Ahari et al. 2020).

**FIGURE 15:**
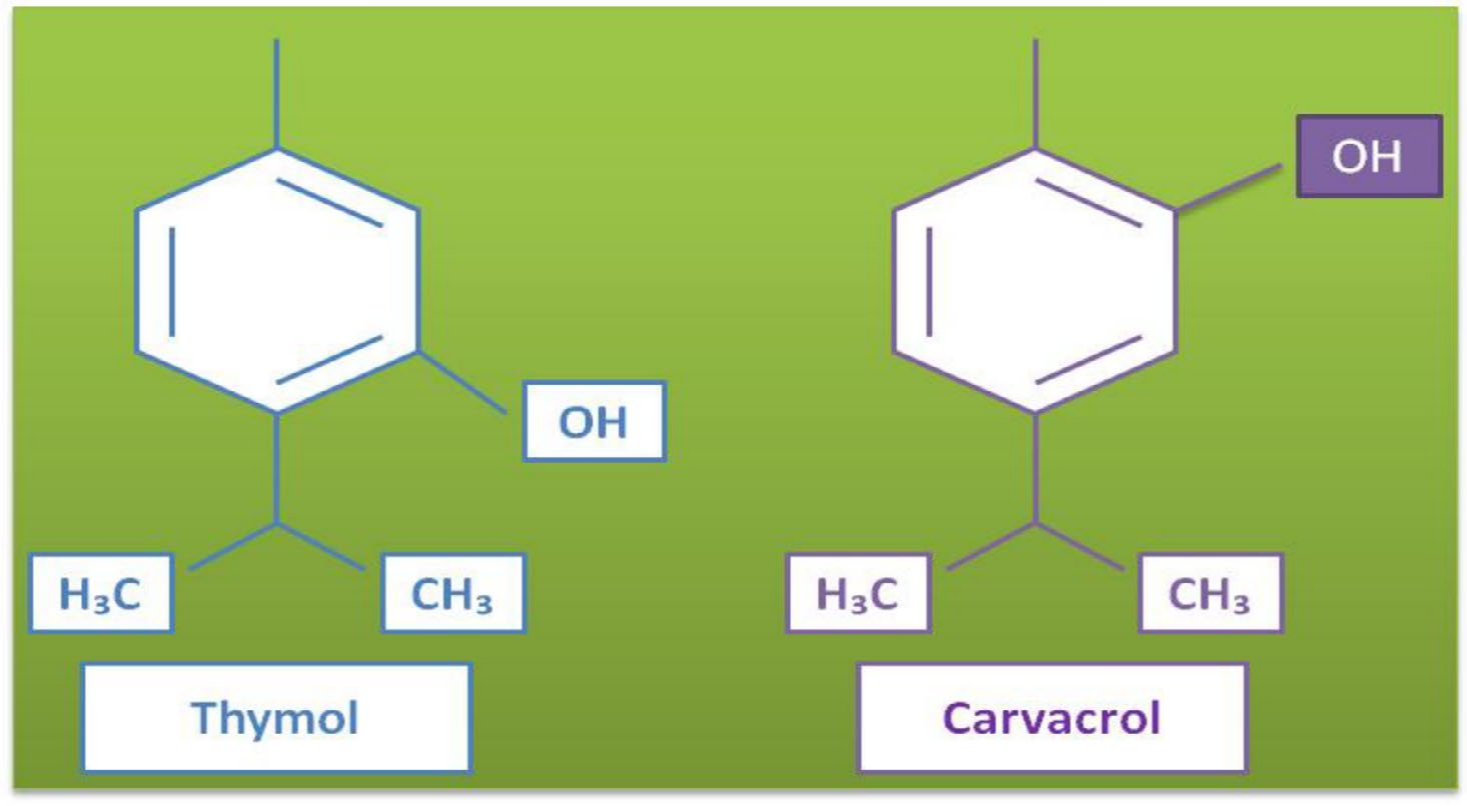
The chemical structures of thymol and carvacrol (Shiyab et al. 2012; Hajibonabi et al. 2023).

Thymus and Origanum, members of Lamiaceae, are the most well‐known thyme species with their unique aromatic flavor and smell. In Turkey, these species are widely used in foods and known as “kekik.” They are utilized in condiments, folk medicine, and as herbal tea. These plants contain EOs, and in general, the major compounds in the essential oils of thymes (THEO) are isomeric phenolic monoterpenes, such as thymol and carvacrol (Figure 15) (Anwar et al. 2025; Hajibonabi et al. 2023; Shiyab et al. 2012). THEO exhibits antimicrobial activities due to its two most well‐known compounds, and therefore, THEO is significant for the food industry, especially for improving the shelf life of food products and inhibiting pathogen microorganisms. In a study, the thyme species growing in Turkey, such as *Thymus sipyleus* subsp. *sipyleus* var. *rosulans*, *Origanum acutidens*, and *O. rotundifolium* were tested with probiotics including *Bifidobacterium bifidum* , *Lactobacillus acidophilus* , *Lactobacillus bulgaricus* , *Lactobacillus plantarum* , *Lactobacillus reuteri* , and *Streptococcus thermophilus* . According to the data, the main compound carvacrol by GC–MS analyses had a great effect on antimicrobial activity. The main compounds in EOs in *Thymus sipyleus* subsp. *sipyleus* var. *rosulans*, *Origanum acutidens*, and *Origanum rotundifolium* are carvacrol (29.99%) and thymol (14.46%), carvacrol (47.46%) and p‐cymene (22.22%), and carvacrol (54.56%) and p‐cymene (12.53%), respectively. *L. bulgaricus* and *S. thermophilus* were the most susceptible bacteria to THEO, while *L. acidophilus* and *L. reuteri* were more resistant than the other strains. As a consequence, THEO can have adverse effects on the microflora in the intestines and especially the qualities of foods produced by fermentation (Cetin et al. 2013).

In conclusion, the EOs of some plants in this study have mostly positive effects on probiotics by inhibiting pathogens, while some of them exhibit a negative influence on probiotic microorganisms. These are summarized in Table 1. In addition to the EOs in some plants shown in Table 1, some plants and their EOs also improve the qualities of several dairy products (Table 2) (Mishra et al. 2020). Moreover, in another study, it was reported that EOs of some plants exhibited significant effects on probiotics and pathogens (Table 3) (Mahmoudi et al. 2017). The probiotics can be used in different lacto‐fermented foods, such as meat products, cereal products, fruits and vegetables, chocolates and candies, etc. So, EOs of some plants can be applied in foods as natural preservatives due to their inhibitory effects on different pathogenic microorganisms (Laranjo et al. 2017).

**TABLE 1:** The effects of EOs of some plants on probiotics.

Plants	Major compounds in EOs	Effects on probiotics	References
Dill ( *Anethum graveolens* )	Carvone, limonene, and α‐phellandrene	Positive	Mohamed et al. (2013), Mehdizadeh et al. (2019)
Chamomile ( *Matricaria recutita* )	(E)‐β‐Farnesene, Germacrene D, and α‐Bisabolol oxide A	Positive	Yangilar and Yildiz (2018), Ahari et al. (2020), Massoud and Sharifan (2020), Al‐Ghanim et al. (2023)
Cinnamon ( *Cinnamomum verum* )	Cinnamaldehyde and eugenol	Positive (especially on *L. rhamnosus* and according to the use of EOs dosage)	Moritz et al. (2012), Valdivieso‐Ugarte et al. (2021), Feltes et al. (2023)
Clove ( *Syzygium aromaticum* L.)	Eugenol, β‐caryophyllene, α‐humulene, and eugenyl acetate	Positive	Moritz et al. (2012), Valdivieso‐Ugarte et al. (2021)
Peppermint (*Mentha piperita* L.)	Menthol and menthone	Positive	Moritz et al. (2012)
Rosemary ( *Rosmarinus officinalis* )	1,8‐cineole, camphor, and α‐pinene	Positive	Massoud and Sharifan (2020)
Chavir (*Ferulago angulata* Boiss.)	α‐pinene and cis‐β‐ocimene	Positive	Keshavarzi et al. (2021)
Zataria ( *Z. multiflora* Boiss.)	Thymol, carvacrol, and *p*‐cymene	Positive	Azizkhani and Tooryan (2016)
Basil (*Ocimum bacilicum*)	Linalool, chavicol, (Z)‐α‐bergamotene, and 1,8‐cineole	Positive	Azizkhani and Tooryan (2016)
Cumin ( *Cuminum cyminum* )	Cumin aldehyde, γ‐terpinene, limonene, α‐ and β‐pinene	Positive ( *B. bifidum* )	Ahari et al. (2020), Mohajeri et al. (2021)
Thyme (*Thymus* and *Origanum* spp.)	Thymol and carvacrol	Negative	Cetin et al. (2013)

**TABLE 2:** The different effects of plants on some dairy products.

EOs of some plants	Dairy products	Effects	References
The EOs of *Zataria multiflora, Ocimum basilicum*, and *Mentha piperita*	Yogurt	Antimicrobial and antioxidant effects	Azizkhani and Parsaeimehr (2018)
The EOs of *Syzygium aromaticum, Salvia rosmarinus*, and *Cinnamomum verum*	Yogurt	Increasing shelf life and antioxidant effect	Azizkhani and Parsaeimehr (2018)
The EOs of *Pimpinella anisum*	Yogurt	Increasing antimicrobial activities	Singh et al. (2011)
The EOs of *Citrus limon, Citrus reticulata*, and *Citrus aurantium*	Ice cream	Enhancing physicochemical, sensorial, and antimicrobial properties	Tomar and Akarca (2019)
The EOs of *Mentha piperita*	Ice cream	Adding functional properties	Yilmaztekin et al. (2019)
The EOs of *Echinophora platyloba*	Cream	Increasing antimicrobial effects and stability	Ehsani et al. (2016)
The EOs of *Satureja cilicica*	Butter	Increasing antioxidant properties and aroma	Ozkan et al. (2007)

**TABLE 3:** The effects of EOs in some plants on probiotics and pathogens.

EOs of some plants on products	Effects on probiotics	Effects on pathogens	References
The EOs of *Cuminum cyminum* L. in yogurt	Positive on *L. casei* , *S. salivarius* , and *L. delbrueckii* subsp. *bulgaricus*	Inhibitory effects on *S. typhimurium*	Mahmoudi (2013)
The EOs of *Mentha longifolia* L. in Iranian white‐brined cheese	Positive on *L. casei*	Inhibitory effects on *S. aureus* and *L. monocytogenes*	Ehsani and Mahmoudi (2013)
The EOs of *Teucrium polium* in yogurt	Positive on *L. casei*	Inhibitory effects on *S. typhimurium*	Mahmoudi et al. (2015)

In general, EOs of some medicinal and aromatic plants can be used for different purposes in foods (Tables 4 and 5). In these applications, some parameters may influence the bioactivity or effectiveness of EOs, like the fat content of meat (Perricone et al. 2015), etc. Therefore, the usage of EOs in foods is combined with innovative packaging methods (Degala et al. 2018). In conclusion, according to the data in Tables 4 and 5, the EOs exhibit antimicrobial effects on pathogens and improve the shelf life of foods, and thus, they can be used as natural preservative agents in various foods, including probiotics.

**TABLE 4:** The usage of EOs in various foods.

EOs of some plants	Foods	Effects	References
The EOs of thyme, grapefruit seed, and lemon mixture	Fish burger	Extending the shelf life of food up to 40%	Saeed et al. (2022)
The EO of oregano	Cod fillets	Exhibiting better results against *P. phosphoreum*	Speranza et al. (2010)
The EO of oregano	Fresh chicken breast meat with modified atmosphere packaging	Keeping the counts of cells below critical levels (7 log CFU/g)	Chouliara et al. (2007)
The EO of *Satureja horvattii*	Pork meat	Effectiveness against *L. monocytogenes* in the preservation of pork for 96 h of storage	Bukvički et al. (2014)
The EOs of lemon grass and ginger	The juice of melon, pear, and apple	Protective against *E. coli* , *Salmonella* spp., and *Listeria* spp.	Raybaudi‐Massilia et al. (2006)
The EO of oregano	Eggplant salad	Impeding the contamination of *E. coli* O157:H7	Saeed et al. (2022)
The EO of oregano	Rice seed	Protective against *B. cereus*	S. A. Burt (2007)
The EO of citrus peel	Bread (applied by spraying it on)	Effective on the sensory characteristics of bread and inhibiting the growth of microorganisms	Saeed et al. (2022)
The EOs of oregano, thyme, and basil	Rice‐based food products	Retarding *B. cereus* activity	Budka and Khan (2010)
The EO of cinnamon (combined with modified atmosphere packaging)	Gluten‐free sliced bread	Extending the shelf life	Gutiérrez et al. (2011)
The EOs of sage, rosemary, anise, and black cumin	Some bakery products	Effective against Gram‐positive and Gram‐negative bacteria, and increases the shelf life of some bakery products	Amany et al. (2012)

**TABLE 5:** Different effects and activities of EOs in some plants on various foods.

Plants	Bioactive compounds in EOs	Effects	References
*Mentha spicata*	Carvone, limonene	Strong antimicrobial activity against *L. monocytogenes* in Lighvan cheese	Moosavy et al. (2013)
*Citrus aurantium*	Limonene	Inhibition of *S. aureus* in sardine fish growth	Djenane (2015)
*Pistacia lentiscus Satureja montana*	β‐Myrcene, carvacrol	Antimicrobial effects on *L. monocytogenes* in minced beef during the storage period	Djenane et al. (2011)
*Zingiber officinale*	α‐Zingiberene, β‐sesquiphellandrene	Inhibition of microorganisms growing in chicken breast filet	Noori et al. (2018)
*Mentha pulegium*	Pulegone, piperitenone	Reducing *L. monocytogenes* growing in Iranian white cheese during storage	Sadeghi et al. (2016)
*Coriandrum sativum*	β‐Linalool	Antibacterial effect on *B. subtilis* in bread	Kačániová et al. (2020)
*Rosmarinus officinalis Thymus vulgaris*	1,8‐Cineole, borneol,p‐cymene, thymol	The reduction effects of their combination on the viable counts of bacteria in vegetables	Iseppi et al. (2019)
*Cymbopogon citratus*	Neral, geranial	More effectiveness on tested bacteria during in vivo and in vitro studies in leafy vegetables	Ramirez et al. (2017)
*Bunium persicum*	Cuminaldehyde	Decreasing the growth of bacteria and extending the shelf life of Iranian white cheese during 45 days of storage time compared to the control samples	Ehsani et al. (2016)
*Cymbopogon citratus*	Geraniol, nerol, myrcene	Controlling the growth of *Aspergillus* species in fermented fish	Dègnon et al. (2019)
*Origanum majorana*	Terpinen‐4‐ol, α‐terpineol	Displaying antifungal and antiaflatoxigenic effects and extending the shelf‐life of maize	Chaudhari et al. (2020)
*Gaultheria fragrantissiuma*	Methyl salicylate	Antifungal and antiaflatoxigenic activities for preservation of millets	Kumar et al. (2018)
*Zingiber officinale*	α‐Zingiberene, geranial	Protection of maize against *A. flavus*	Nerilo et al. (2020)
*Origanum vulgare Eugenia* spp.	Carvacrol, eugenol	Both exhibit antifungal activity and maintain a sensory profile at the same time in salad	Ribes et al. (2019)
*Myristica fragrans*	Elemicin, myristicine, thujanol	Preserving rice against fungal infestation and aflatoxin B1 secretion	Das et al. (2020)
*Ocimum gratissimum*	Methyl cinnamate, ɣ‐terpinene	Controlling fungal‐ and aflatoxin‐mediated biodeterioration in spices	Prakash et al. (2011)

## 4 Conclusion

The findings presented in this review highlight the multifaceted role of EOs in lacto‐fermented food systems, particularly in shaping microbial communities, influencing fermentation processes, and contributing to the overall quality and stability of dairy products. EOs have broad‐spectrum antimicrobial properties; their impact on lactic bacteria and pathogenic microorganisms remains complex and context‐dependent. Studies have shown that certain EOs can enhance or inhibit the survival and metabolic activity of probiotic strains in fermented foods. While lower concentrations of *Lactobacillus* and *Bifidobacterium*‐friendly EOs, such as *O. basilicum* (basil) and *R. officinalis* (rosemary), may support bacterial viability by modulating oxidative stress and cell membrane integrity, higher concentrations of phenol‐rich EOs, including *O. vulgare* (oregano) and *Thymus vulgaris* (thyme), have been reported to exert inhibitory effects, reducing probiotic populations. The delicate balance between probiotic survival and EO concentration suggests the necessity for precise formulation strategies in fermented food applications.

EOs demonstrate significant efficacy in suppressing foodborne pathogens, with *Cinnamomum zeylanicum* (cinnamon), *Syzygium aromaticum* (clove), and *O. vulgare* (oregano) emerging as the most potent inhibitors of *Listeria monocytogenes* , *E. coli* , *S. aureus* , and *Salmonella* spp. These EOs act primarily through membrane disruption, ion leakage, and inhibition of enzymatic activity, leading to compromised cellular function in pathogenic bacteria. However, variations in the MICs across different strains highlight the need for strain‐specific evaluations to optimize their antimicrobial potential in fermented food matrices. Beyond their antimicrobial effects, EOs influence key sensory and textural attributes of fermented dairy products. Compounds such as linalool, eugenol, and thymol contribute to aroma complexity and flavor enhancement, yet excessive concentrations may introduce undesirable bitterness or pungency. Additionally, EOs interact with the protein and fat matrix of dairy products, potentially affecting coagulation properties and mouthfeel. Studies indicate that controlled EO incorporation can extend shelf life by delaying lipid oxidation and proteolytic degradation, thereby improving product stability and consumer acceptability.

The potential of EOs as natural preservatives in the food industry, particularly in the production of fermented foods, remains a promising yet challenging avenue. Their ability to replace synthetic preservatives aligns with consumer demand for clean‐label products; however, formulation challenges, such as EO volatility, hydrophobicity, and dose‐dependent effects on fermentation dynamics, require further research. Encapsulation techniques, nanoemulsions, and carrier‐based delivery systems have been used to improve EO's stability and bioavailability while minimizing adverse effects on probiotic viability. Despite the substantial body of evidence supporting the antimicrobial and quality‐enhancing properties of EOs, several limitations persist in the existing research. Many studies focus on in vitro conditions, which may not completely replicate the complex biochemical interactions in real food matrices. Additionally, the determination of optimal EO concentrations that ensure antibacterial activity without compromising probiotic growth remains an area requiring more rigorous investigation.

To address these gaps, future research should explore:

### 4.1 Optimization of EO Concentrations

Establishing precise EO concentrations that balance antimicrobial efficacy with probiotic preservation in fermented food systems.

### 4.2 Synergistic Effects of EOs

Investigating the combined effects of multiple EOs to enhance antimicrobial activity while reducing individual EO concentrations to minimize adverse sensory impacts.

### 4.3 Encapsulation and Controlled Release Strategies

Developing innovative EO delivery methods that improve stability, control release rates, and maintain the sensory integrity of fermented foods.

### 4.4 In Vivo and Industrial Scale Studies

Expanding research beyond laboratory settings to validate findings in large‐scale food production environments and assess the commercial feasibility of EO‐based preservation strategies.

In conclusion, while EOs hold great potential as natural preservatives in lacto‐fermented food systems, their successful application requires careful optimization to balance antimicrobial efficacy, probiotic viability, and sensory acceptability. Addressing current research limitations through interdisciplinary approaches will be key to unlocking their full potential in the food industry.

## Author Contributions

**Ibrahim Canbey:** investigation (equal), methodology (equal), writing – original draft (equal). **Tulay Ozcan:** conceptualization (lead), investigation (equal), writing – original draft (equal), writing – review and editing (lead). **Ozan Gurbuz:** investigation (equal), project administration (equal).

## Ethics Statement

This study does not involve any human or animal testing.

## Conflicts of Interest

The authors declare no conflicts of interest.

## Data Availability

Data will be made available upon reasonable request.
